# Autistic traits modulate neural and behavioral responses to social vs nonsocial rewards

**DOI:** 10.1017/pen.2025.10003

**Published:** 2025-09-04

**Authors:** Anthony Haffey, Chun-Ting Hsu, Bhismadev Chakrabarti

**Affiliations:** 1 Centre for Integrative Neuroscience and Neurodynamics, School of Psychology and Clinical Language Sciences, University of Reading, Whiteknights Campus, Reading RG6 6AL, UK; 2 Department of Psychology, Ashoka University, Sonipat, India; 3 India Autism Center, Kolkata, India

**Keywords:** autism, connectivity, MRI, Psychophysiological interaction (PPI)

## Abstract

Social rewards (e.g. smiles) powerfully shape human behavior, starting from early childhood. Yet, the neural architecture that enables differential processing of social and nonsocial rewards remains largely unknown. Few previous studies that directly compared social vs nonsocial stimuli have used stimuli that have low ecological validity or are not matched on low-level stimulus parameters – limiting the scope of inference. To address this gap in knowledge, social and nonsocial reward images taken from the real world were matched on valence, arousal, and key low-level stimulus properties and presented to 37 adults in a functional magnetic resonance imaging (fMRI) study. Individual self-reported preference for social images was associated with the functional connectivity between the left anterior insula (LAI) and medial orbitofrontal cortex (mOFC), as well as that between the left Fusiform Gyrus (LFG) and the Anterior Cingulate Cortex (ACC). Autistic traits negatively modulated LAI – mOFC connectivity and LFG – ACC connectivity. Reduced functional connectivity between these regions may contribute to the lower social reward responsivity in individuals with high autistic traits, as also noted from their lower valence ratings to social rewards. This study provides evidence for a new experimental paradigm to test social reward processing at a behavioral and neural level, which can contribute to potential transdiagnostic biomarkers for social cognitive processes.

## 1. Introduction

Social rewards such as smiles are crucial components of social interactions, act as powerful reinforcers, and help shape human behavior and create rapport. Studies in young infants have shown how most children prefer to orient to a face/voice compared to comparable nonsocial stimuli (Morton & Johnson, 1991; Vouloumanos et al., 2010). Studies in adults have shown a similar preference for social over nonsocial rewards (Chakrabarti et al., 2017; Fletcher-Watson et al., 2008). The preference for social over nonsocial rewards has been tested extensively using behavioural and eye-tracking paradigms (Hedger et al., 2020; Frazier et al., 2017). In contrast, comparatively few studies have directly investigated the neural processing of social vs nonsocial rewards.

The reward system is one of the most-studied networks of brain regions. Insights from electrophysiological studies in rodent and non-human primates as well as from functional magnetic resonance imaging (fMRI) studies in humans have revealed the importance of a few key nodes in this network, that include the medial orbitofrontal cortex (mOFC), ventral striatum (VS), and amygdala (Haber & Knutson, 2010). There are fiber tracts between the VS and the mOFC (Haber et al., 2006; Lehéricy et al., 2004). The VS also receives input from the amygdala (Friedman et al., 2002; Fudge et al., 2002; Russchen et al., 1985) in primates, which plays a key role in tagging salience of emotional (Anderson & Phelps, 2001) and rewarding stimuli (Mahler & Berridge, 2009). The majority of studies on the neuroanatomy of reward processing comes from the processing of nonsocial rewards such as food, chocolate, or money.

Studies that have explored social reward processing have found social rewards to activate a similar set of regions to those described earlier (Fareri, Chang, & Delgado, 2012; Fareri, Chang, & Delgado, 2015; Izuma et al., 2008; Lin, Adolphs, et al., 2012; Wake & Izuma, 2017; Zink et al., 2008). The VS, mOFC, and amygdala have been shown to respond to social rewards, including smiles (Scott-Van Zeeland et al., 2010), positive feedback (Izuma et al., 2008), cooperation (Elliott et al., 2006), social status (Zink et al., 2008), charitable giving (Kuss et al., 2013), and interacting with friends (Güroğlu et al., 2008). In an early study, mOFC activity in response to monetary reward was increased by co-operation to win this money (Elliott et al., 2006), suggesting that this activity could constitute a common neural currency for both social and nonsocial rewards (Grabenhorst et al., 2010). More recently, the temporoparietal junction (TPJ) has been shown to modulate social value processing in the ventromedial prefrontal cortex (Strombach et al. 2015). In addition, the anterior cingulate cortex (ACC) responds to rewards and punishment for conspecifics, supporting its role in social reward processing (Schneider et al., 2020).

Most studies mentioned above presented social rewards in isolation, which do not allow for a direct comparison of social and nonsocial reward processing within the same experimental paradigm (but see Rademacher et al., 2014; Scott-Van Zeeland et al., 2010 for notable exceptions). In recent years, a class of paradigms comparing social vs nonsocial rewards directly involves the use of the monetary incentive delay and the social incentive delay task. (Gu et al., 2019). This paradigm requires participants to make and remember associations between shapes/ symbols with reward outcomes and primarily indexes the anticipatory phase of reward processing. In contrast, paradigms directly presenting images of social and nonsocial rewards are likely to primarily index the consummatory phase of reward processing. In the minority of studies that formally compared responses to images of social vs nonsocial rewards, the stimuli either have low ecological validity (e.g. point-light figures) or are not matched for their low-level stimulus properties – which poses challenges for interpretation (Saygin et al., 2004; Sasson et al., 2008).

Differences in individual responsivity to social rewards exist across the population but can be particularly notable in conditions such as autism. Autistic individuals often show reduced responsivity to social rewards in lab-based assessments (Hedger et al., 2020). Lower activity in reward-related brain regions such as mOFC and VS to social rewards has also been reported in autistic compared to non-autistic adults (Kohls, Schulte-Rüther, et al., 2013; Scott-Van Zeeland et al., 2010) but see Dichter et al. (2012) for an exception. A meta-analysis of neuroimaging studies reported differential reward system responses to both social and nonsocial rewards in autistic individuals (Clements et al., 2018). Similar to the studies on non-autistic individuals, the majority of studies included in this meta-analysis examined social or nonsocial reward processing in isolation.

To address the gaps in the literature, the current study aimed to develop and test a new stimuli set and paradigm to investigate neural processing of social vs. nonsocial rewards, when these different categories of stimuli are closely matched on stimulus parameters. The stimuli were drawn from well-characterized real-world images and hence have higher ecological validity than schematic faces/ posed expressions. However, it is worth noting that all the pictures may not evoke strong subjective feelings of reward. To this end, our use of the term “reward” is more aligned with the definition of emotion proposed by Ralph Adolphs – in proposing that it is a functional state that can enable subjective feelings of pleasure, as well as behavioral changes (Fox et al., 2018). A secondary aim was to investigate if individual differences in functional connectivity between key brain nodes involved in reward and social processing was associated with autistic traits. In light of the previous studies on autism and social rewards, we hypothesized that individuals with high autistic traits will have reduced preference for social rewards, and have reduced functional connectivity between brain regions involved in social reward processing (Chevallier et al., 2012)

## 2. Materials and methods

### 2.1. Participants

40 participants completed the study from the local population in and around the university. Two participants were excluded from the analysis due to a programming error and one participant was removed due to excessive movement (see *data analysis*), leaving 37 participants (19 females; mean age = 22.89 years, S.D. = 5.02 years, min = 18, max = 40). All participants had normal or corrected to normal vision and completed the 50-item Autism Spectrum Quotient with binarized response format (AQ; Baron-Cohen et al., 2001). The mean AQ score was 14.3 (SD = 7.74), the scores ranged from 3–33 and a higher score reflects more autistic traits. Participants were not asked about whether they had a clinical diagnosis of any neurodevelopmental conditions, such as autism. Autistic traits are continuously distributed between those with and without a diagnosis (Robinson et al., 2011).

### 2.2. Design and materials

Forty pairs of social and nonsocial reward images (images can be requested from the authors) were taken from the International Affective Picture System (Lang et al., 1999) and downloaded from the website Flickr. Social reward images included one or more humans (e.g. happy individuals), while nonsocial reward images included rewarding nonsocial objects (e.g. food and scenery). The social images comprised happy children and adults in different real-world contexts ranging from weddings, sports events, social events, and family photographs. Half of the nonsocial images included food, and the remaining images were of scenery, vehicles, money, and one piece of jewellery.

The images were modified with ImageJ (Abràmoff, 2004) and Adobe Photoshop to match them for size and multiple image parameters (e.g. contrast and luminance). The Koch toolbox (Walther & Koch, 2006) was used to calculate image saliency for each pair of images in a previous paper, and there was no mean difference in luminosity, root mean square (RMS) contrast (local and global), as well as Koch toolbox metric of image saliency between social and nonsocial images (Chakrabarti et al., 2017). Previous studies using these stimuli have demonstrated greater gaze fixation for social over nonsocial images using eye-tracking, and quicker subjective awareness of social over nonsocial stimuli using continuous flash suppression (Chakrabarti et al., 2017; Gray et al., 2018; Hedger et al., 2018, 2020).

All stimuli were displayed using E-prime 2.0 (Psychology Software Tools, PA, USA) on a ViewSonic VE510s monitor (ViewSonic Corporation, California, USA).

### 2.3. Procedures

Participants performed a rating task outside the scanner and a fMRI task. During the rating task, participants were presented with an image to give a valence rating on a 9-point Likert scale using the manikins from (Bradley & Lang, 1994). Participants were instructed to rate how happy the images were by clicking one of 9 manikins conveying a range of emotions from unhappy to happy.

During the fMRI task, the social and nonsocial images were presented in pseudo-randomized blocks of four images of the same condition (social/ nonsocial), followed by a block of four subsequent fixation crosses. The order of presentation of social and nonsocial images was randomized within these constraints. Each image was presented for 4 seconds, fixation crosses for 3 seconds, followed by 1 second of blank screen. To maintain attention to the task, participants were required to press a button with their right index finger during each presentation of an image or fixation cross. The 50-item Autism Quotient questionnaire (AQ; Baron-Cohen et al., 2001) was completed online after the scanning session.

### 2.4. Valence ratings

Across the whole sample, t-tests were used to test a) whether the images are perceived as rewarding and b) that social images were not significantly different in their reward value to nonsocial images. At an individual level, mean valence ratings were calculated separately for social reward images and nonsocial reward images. Pearson’s r was computed for correlations between AQ and these mean ratings to test whether autistic traits were associated with diminished reward value for social rewards. Additionally, a composite metric for quantifying individual preference for social vs. nonsocial rewards (i.e. ***sociality bias***) was computed by subtracting nonsocial from social mean valence ratings. Valence ratings that were 3 or more SDs away from the mean across both social and nonsocial images were removed as outliers for each participant.

### 2.5. Regions of interest

**Figure 1.**
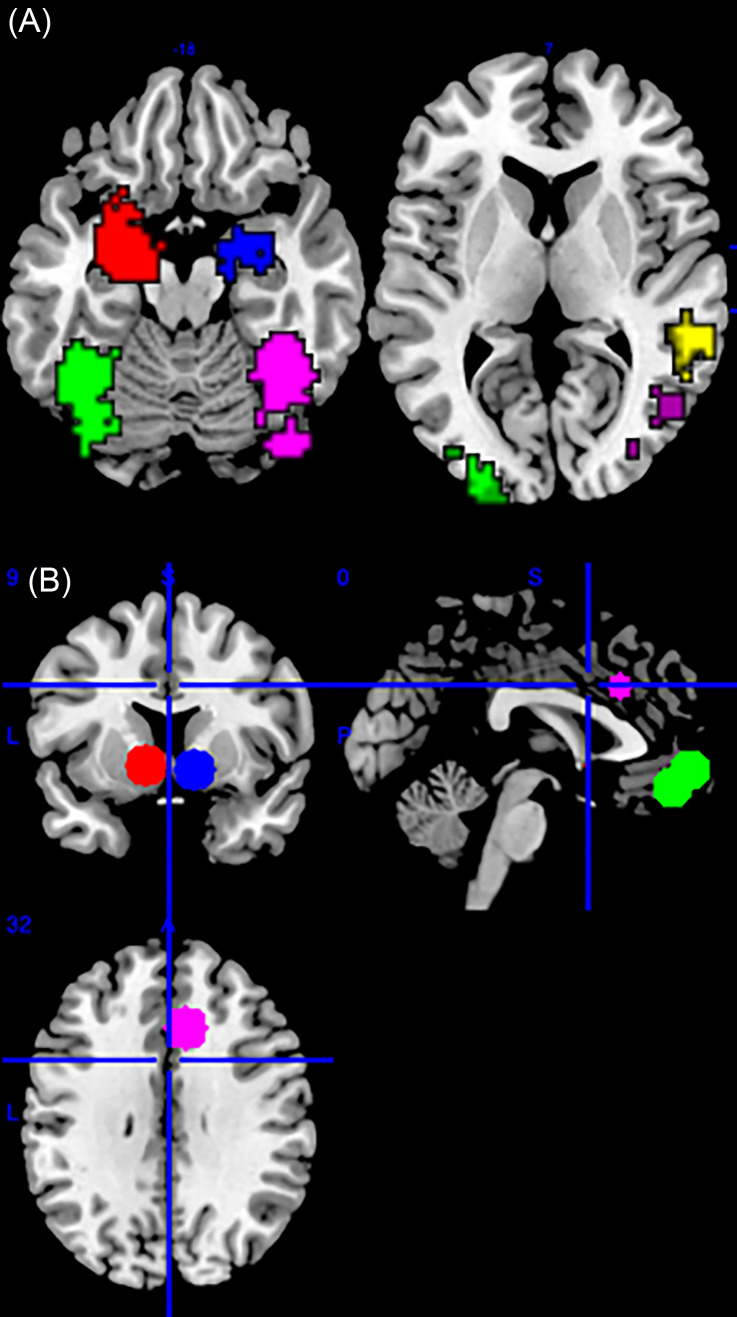
Predefined regions of interest. A. Axial sections at MNI *z* = –18 (left) and *z* = 7 (right) showing ROIs defined functionally by the activation maps of Atzil et al.’s multilevel peak kernel density analysis: left amygdala (red), right amygdala (blue), left FG (green), right FG (magenta), and right TPJ (yellow). B. Sections at MNI *x* = 0 (upper right), *y* = 9 (upper left), and *z* = 32 (lower) showing ROIs defined functionally by making 10 mm spheres centered on coordinates of brain areas activated by reward outcome reported in Liu et al.: left VS (red) and right VS (blue)], mOFC (green), and ACC (magenta).

Based on our a priori hypotheses, we selected our specific regions of interest (ROIs) based on meta-analyses of sociality- (Atzil et al., 2023) and reward-related (Liu, et al., 2011) brain regions. The masks for sociality-related regions were defined functionally by the activation maps of Atzil et al.’s multilevel peak kernel density analysis on the contrast: [All social stimuli > No social stimuli] (bilateral amygdala, bilateral FG, and right TPJ, Fig. 1A). The masks for reward-related regions were defined functionally by making 10mm spheres centered on coordinates of brain areas activated by reward outcome (bilateral VS: [12, 10 −6] and [−10 8 −4], and one mask made of two overlapping spheres of bilateral mOFC: [−2 56 −6] and [2 48 −14], and ACC [8 24 32], Fig. 1B) reported in Liu et al.

### 2.6. fMRI data acquisition and preprocessing

Scans were conducted using a 3T Siemens TIM Trio MRI scanner with 12-channel head coil. One run of 322 volumes was measured per participant using a T_2_*-weighted echo-planar sequence [slice thickness: 3 mm, no gap, 37 slices, repetition time (TR): 2s, echo time (TE): 30ms, echo spacing: 0.53ms, generalized autocalibrating partially parallel acquisitions (GRAPPA) acceleration factor: 2, flip angle: 90°, matrix: 64 × 64, field of view (FOV): 192 mm, voxel size: 3.0 mm × 3.0 mm × 3.0 mm] and individual high-resolution T1- weighted anatomical data (MPRAGE sequence) were acquired (TR: 2.2, TE: 2.9, FOV: 250, matrix: 256 × 256, sagittal plane, slice thickness: 1 mm, 176 slices, resolution: 1.0 mm × 1.0 mm × 1.0 mm). The phase and magnitude images for the construction of the field map were collected with the same positions and dimensions as the functional scans [slice thickness: 3 mm, no gap, 32 slices, TR: 400 ms, TE: 5.19 and 7.65 ms, flip angle: 60°, matrix: 64 × 64, FOV: 192 mm, voxel size: 3.0 mm × 3.0 mm × 3.0 mm].

The data were then further preprocessed and analyzed using MATLAB 2024b and the software package SPM25 (www.fil.ion.ucl.ac.uk/spm). Preprocessing consisted of slice-timing correction, realignment for motion correction, unwarping with the field map in the first and second order for rotations along the x and y axis, and coregistration of the structural image to the mean realigned functional image. Structural images were segmented into gray matter, white matter, cerebrospinal fluid, bone, soft tissue, and air/background with the “New Segment” module (Ashburner & Friston, 2005). A group anatomical template was created with DARTEL (Diffeomorphic Anatomical Registration using Exponentiated Lie algebra; Ashburner, 2007) toolbox from the segmented gray and white matter images. Transformation parameters for structural images were then applied to functional images to normalize them to the brain template of the Montreal Neurological Institute (MNI) supplied with Statistical Parametric Mapping (SPM). Functional images were spatially smoothed with a kernel of 6 mm full-width-at-half-maximum after normalization.

### 2.7. fMRI mass univariate GLM analysis

Two participants were removed due to programming error: blocks were presented in an entirely random order to the first two participants, resulting in clusters of 5 blocks of the same stimulus type in a row for both. The block order of the remaining participants was pseudo-randomized so that half the participants received one order of social and nonsocial blocks, whereas the other received the reversed order. Due to the difference between the block structure in the first two participants and the remaining 38, these two participants were not included in the analysis. One further participant’s data was removed due to excessive movement, having moved more than 3 mm between two volumes, leaving 37 participants.

Statistical parametric maps were calculated with multiple regressions of the data onto a model of the hemodynamic response (Friston et al., 1995). The first level general linear model analyses contained two psychological regressors of interest for “Social” and “Nonsocial” conditions in the design matrix. Each block in each condition lasted 16 seconds. Psychological regressors were convolved with the canonical hemodynamic response function. Six motion parameters were included as nuisance regressors. A high-pass filter with a cut-off period of 128 s was applied, and the temporal autocorrelation was accounted for with the AR(1) option. For each ROI, the first eigenvariate of the contrast [social – nonsocial] for each participant were extracted with MarsBaR (version 0.45).

### 2.8. fMRI mass univariate PPI analysis

In order to test whether the sociality-and reward-associated brain activity in ROIs is functionally coupled, a generalized Psychophysiological Interaction analysis (gPPI toolbox v13.1; Friston et al., 1997; McLaren et al., 2012) was performed. ROI’s from Atzil’s et al. (2023) were defined as the seed region, while ROIs from Liu et al. (2011) others were defined as target regions. The vector of neural response was estimated by deconvoluting the first eigenvariate of the BOLD signal extracted from the seed region. The interaction vector of each condition was calculated as the product of the estimated neural response vector and the condition vector’s ON times. We then performed the general linear model analysis for the whole brain, including the interaction vectors, the condition vectors, and the estimated neural response vector of the seed region as regressors. For each target region, the first eigenvariate of the univariate contrast between the interaction term of the social condition and that of the nonsocial condition for each participant were extracted with MarsBaR (version 0.45) and used for the group-level one-sample t-test. To test whether autistic traits modulated the functional connectivity between brain activity associated with social stimuli processing and reward value responsiveness, AQ was correlated with the contrast values of the target regions in the gPPI analysis.

For the purposes of completeness, we provide whole-brain analyses in the *supplementary materials.* Family-wise error corrected p < 0.05 at the peak level, cluster size ≧ 5, was used as the significant threshold for whole-brain results.

Participants were identified as correlational outliers if their Cook’s D value was greater than 4/35 (i.e. 4/(N-1-K)) and were removed from the specific correlation that they were outliers for.

All analyses use 1-tailed p-values to reflect the directional nature of the hypotheses that: autistic traits will be associated with reduced sociality bias and reduced connectivity between social and reward regions; sociality bias will be associated with increased connectivity between social and reward regions. Bootstrapped confidence intervals with replacement based on 10,000 repetitions are included for all correlations to address concerns about small sample sizes. Holm–Šidák corrections for multiple testing are applied to correlations between *sociality bias* and connectivity for each pair of regions (see supplementary materials 2).

## 3. Results

### 3.1. Rating task

T-tests of the mean ratings of the images were conducted to test the assumption that the images were similarly pleasant to participants in this sample. No significant difference between social and nonsocial was found across the whole sample (*t*(36) = .47, *p* = .643, 95% CI [−0.3, 0.18], *d* = .08). One sample t-tests found that the mean ratings for social (M = 6.47, SD=.69, *t*(36) = 13.01, *p < .*001, 95% CI [6.24, 6.69], *d* = 2.14) and nonsocial images (M=6.52, SD=.63, *t*(36) = 14.66, *p < .*001, 95% CI [6.31, 6.73], *d* = 2.41) were significantly above the neutral rating of 5, suggesting that the participants found the images rewarding. Against expectations, some participants rated some images as unpleasant (less than 5), but this was only true for 5.45% of social images and 4% of nonsocial images across all participants.

**Figure 2.**
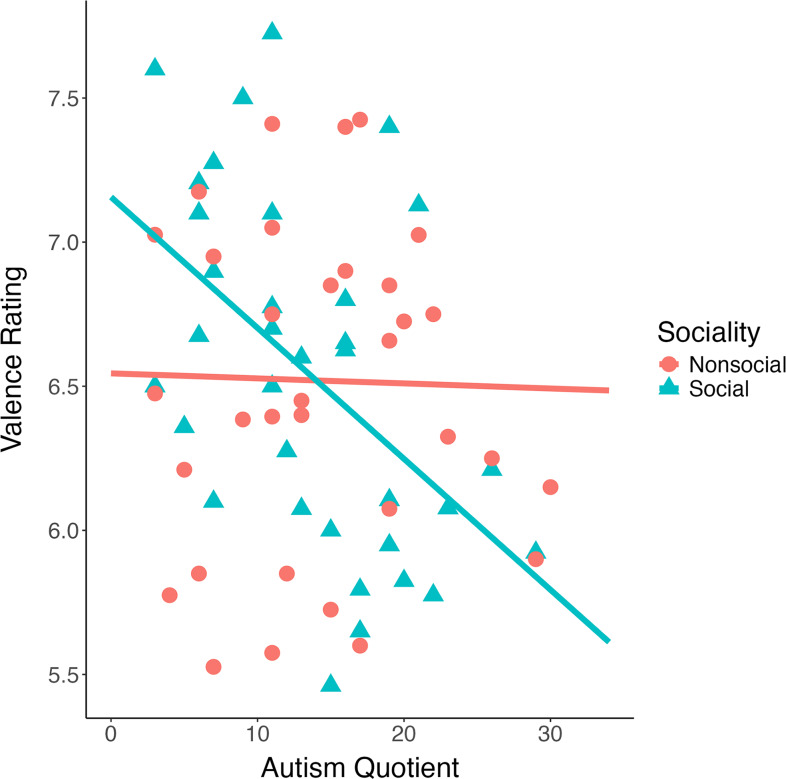
Scatterplot of associations between autistic traits measured using the Autism Spectrum Quotient (AQ) and social and nonsocial valence ratings. *Note:* These results visualize how autistic traits (AQ) are negatively associated with valence ratings for social (*r*(32) = –.49, *p* = .001) but not nonsocial images (*r*(33) = –.02, *p* = .9).

AQ correlated negatively with valence ratings of social rewards (*r*(32) = −.49, p = .001; bootstrap CI: [−.68; −.3]) but no such pattern was seen for valence ratings of nonsocial rewards (*r*(33) = −.022, p = .901; bootstrap CI: [−.26; .23]). The negative association between AQ with *sociality bias* was significant (*r*(35) = −.36, *p* = .016; bootstrap CI: [−.54; −.19]), consistent with autistic traits being associated with reduced preference for social than nonsocial images (see Fig. 2).

### 3.2. Brain data

#### 3.2.1. Whole-brain GLM results

At the whole brain level, the following regions showed significantly stronger activity in the social than in the nonsocial condition: bilateral amygdala, right fusiform gyrus, right medial orbitofrontal cortex (gyrus rectus), and bilateral temporo-occipital junction. Bilateral fusiform gyrus, visual cortex, and left lateral frontopolar cortex were more active in the nonsocial than in the social condition (Table 1). No significant result was found in the whole-brain correlation of the social-nonsocial contrast values with AQ or social bias.

**Table 1.** Whole brain results of Social vs. Nonsocial conditions

H	Regions	Cluster size	***p*** (FWE-corr)*	***T***	B.A.	**[** ***x***, ***y***, ***z*** **]**
**Social > Nonsocial**						
R	Inferior occipital	404	<.001	15.56	37	51, –72, 3
	Middle temporal		<.001	12.13	37	48, –63, 12
L	Middle occipital	285	<.001	11.31	19	–45, –75, 9
	Middle occipital		<.001	10.97	19	–51, –78, 0
R	Fusiform	53	<.001	9.63	37	39, –48, –18
R	Amygdala	13	<.001	7.31	–	18, –6, –15
R	Superior temporal	18	0.001	7.17	21	51, –9, –15
R	Gyrus rectus	11	0.001	7.16	11	3, 54, –21
R	Temporal pole	10	0.001	7.07	20	42, 12, –36
L	Amygdala	3	0.004	6.49	–	–18, –6, –15
**Nonsocial > Social**						
L	Fusiform	88	<.001	11.37	37	–27, –48, –18
L	Middle occipital	61	<.001	8.12	19	–30, –87, 12
R	Fusiform	59	<.001	8.00	37	27, –48, –15
R	Middle occipital	27	0.001	7.11	18	30, –81, 9
L	Anterior orbital gyrus	9	0.001	7.05	11	–24, 39, –15

* Voxel level FWE-corrected for the whole brain.

Abbreviations: H = hemisphere; L = left; R = right; p = p-value; T = T-value; B.A. = Brodmann area; x, y, z = MNI coordinates.

#### 3.2.2. Brain-behavior relationship

After applying Holm–Šidák corrections to alpha thresholds to address multiple testing, *sociality bias* was positively associated with functional connectivity from the LFG to ACC (r(30) = .64, p < .001; bootstrap CI: [.36; .78]) and the LAI to mOFC (r(34) = .52, p < .001; bootstrap CI: [.35; .66]). AQ was significantly associated with both LFG to ACC (r(31) = −.3, p = .04; bootstrap CI: [−.54; −.05]) and LAI to mOFC connectivity (r(32) = −.45, p = .004; bootstrap CI: [−.67; −.15]).

**Figure 3.**
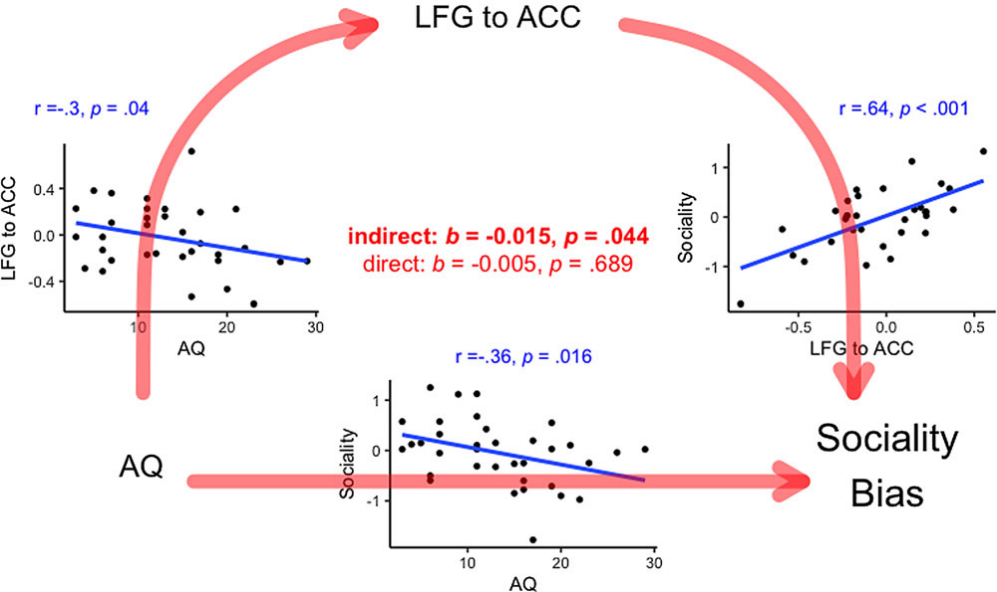
Grid of scatterplots summarizing the associations between AQ, LFG to ACC connectivity and sociality bias (in blue), with the indirect and direct from mediation analysis (in red). *Note:* all correlations are significant under non-parametric Spearman’s rank correlation (see supplementary analysis 1).

**Figure 4.**
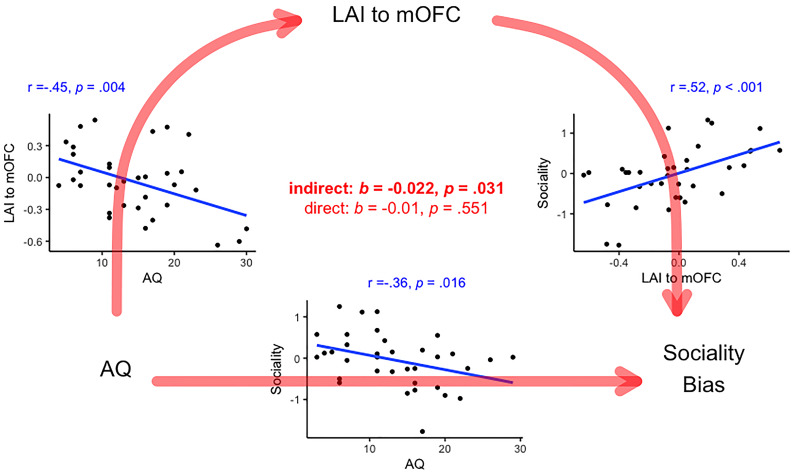
Grid of scatterplots summarizing the associations between AQ, LAI to mOFC connectivity and sociality bias (in blue), with the indirect and direct from mediation analysis (in red). *Note:* all correlations are significant under non-parametric Spearman’s rank correlation (see supplementary analysis 1).

Mediation analysis found that LFG to ACC connectivity fully mediated the association between AQ and *sociality bias* as the indirect effect was significant (*b* = −.015, *p* = .044, 95% bootstrapped confidence interval lower limit/LL = −.035, 95% bootstrapped confidence interval upper limit/UL < −.001) but the direct effect was not significant (*b* = −.005, *p* = .689, bootstrapped LL = −.035, UL = .019; See Fig.3). Mediation analysis found that LAI to mOFC connectivity fully mediated the association between AQ and *sociality bias* as the indirect effect was significant (*b* = − .022, *p* = .031, bootstrapped LL = −.049, UL = −.002) but the direct effect was not significant (*b* = −.01, *p* = .551, LL = −.056, UL = .022; See Fig.4).

## 4. Discussion

We compared response to social vs nonsocial rewards in a sample of young adults using self-report and fMRI. We found that behavioral preference for social over nonsocial rewards (i.e. sociality bias) was positively associated with functional connectivity of LFG-ACC and LAI-mOFC. We further found that the relationship between autistic traits and sociality bias is fully mediated by the functional connectivity of LFG-ACC and LAI-mOFC.

A large number of behavioral and eye-tracking studies have previously compared processing of social vs nonsocial stimuli (Hedger et al., 2020; Frazier et al., 2017). Most of these studies have been conducted in young children, and included both rewarding and neutral stimuli. The few studies that have directly compared social and nonsocial reward processing within a single paradigm have tested the anticipatory component of reward processing rather than the consummatory component of it (Ait Oumeziane et al., 2017; Lin, Adolphs, et al., 2012; Lin, Rangel, et al., 2012; Spreckelmeyer et al., 2009; Wake & Izuma, 2017; although see Rademacher et al., 2010). The current study focuses on the consummatory component of reward processing, by presenting the social and nonsocial reward stimuli to the participants without them having to perform any action. The current design thus allows us to infer the differential neural response to receipt of these different types of reward, without the impact of trade-offs between effort minimization and value maximization. Additionally, the close matching of the social and nonsocial reward stimuli allows us to make inferences that are less subject to confounds due to stimulus features such as contrast, luminosity, and saliency.

We found that even when social reward images are matched on basic stimulus properties, and rated as equally rewarding to nonsocial reward images across the full sample, autistic traits were coupled with individual preference for social rewards both behaviorally and neurally. At a behavioral level, autistic traits were inversely proportional to the valence ratings for social reward images. This relationship was not seen for nonsocial rewards. This observation is consistent with earlier empirical reports showing a reduced rating of social stimuli or situations by autistic individuals in lab-based studies (Chevallier et al., 2012; Sasson et al., 2012). At the neural level, we found that the functional coupling of LFG-ACC and LAI-mOFC was weaker in individuals with higher autistic traits. While the role of the fusiform gyrus in conspecific face and body processing is well established, a recent study has also identified it as a hub of expression for genes involved in synaptic transmission as well as autism (Chen et al., 2022). Neurochemically, an earlier study reported reduced GABA_B_ receptors in both fusiform gyrus as well as the ACC – which are involved both in maintaining synapses and the excitation-inhibition balance in the brain (Oblak et al., 2010). Functionally, a weaker coupling between fusiform gyrus and anterior cingulate cortex corresponds to reduced links between social perceptual encoding and value encoding. Assuming a certain degree of functional specialization in the brain, the fusiform gyrus and nearby areas are more involved in perceptual encoding of stimuli (Naspi et al., 2021) – while the ACC is arguably involved more in encoding stimulus value (Cai and Padoa-Schioppa, 2012). This observation is also consistent with earlier accounts of reduced links between reward and social processing in autism (Neufeld et al., 2019; Sims et al., 2014).

The LAI-mOFC functional coupling represented another link between social perceptual and value encoding, which we found to be negatively modulated by autistic traits and further modulate social bias. Anterior insula receives interoceptive and nociceptive afferent input from inner organs and skin (Craig, 2009), and has long been associated with pain empathy (Lamm et al., 2007, 2009, Bernhardt and Singer, 2012) and social norm compliance (Bellucci et al., 2018). Previous studies have investigated the role of interoception in social cognition (Arnold et al., 2019). For example, higher interoceptive awareness is associated with less negative affect after challenging social situations, due to the ability to properly attribute the physiological response as resulting from the external social situation, resulting in better emotional regulation in social situations (Werner et al., 2013). The mOFC is associated with reward value encoding (Kringelbach, 2005; Rolls et al., 2020). In mammals, there is direct projection from anterior insula to orbitofrontal cortex (Mathiasen et al., 2023). In humans, the coactivation of LAI and mOFC has been associated with the pro-social behavior of self-impression/reputation management when being observed by others (Yoon et al., 2021), which was said to be a behavioral manifestation of allostatic regulatory process, to prevent homeostatic disturbances accompanied by social threat/stress/punishment (Slavich et al., 2010). In the present study, participants showing preference for social rewards had enhanced functional coupling when viewing social stimuli with human figures, which might be associated with stronger incentive or preparedness for self-impression/reputation management and the consequential social rewards (Tennie et al., 2010).

Interestingly, the functional coupling of LFG-ACC and LAI-mOFC fully mediated the association between AQ and the behavioral preference for social over nonsocial rewards (i.e. *sociality bias*). While autism has been associated with reduced preference for social rewards in several prior studies (Frazier et al., 2017; and specifically using these stimuli by Hedger et al., 2018), direct investigations of underlying mechanisms linking neural and behavioral data have been rare. To this end, the mediation analysis suggests an indicative neural mechanism to explain the behavioral observation of reduced preference for social rewards in individuals with high autistic traits. *However,* similar to correlations, claims about causality from mediation in cross-sectional data are more vulnerable to overlooking causal covariates or confounds than experimental or longitudinal designs (Rohrer et al., 2022).

Finally, we discuss the results of the whole-brain univariate contrast for the sake of completeness. These analyses (Table 1) showed that social condition more specifically activated the right fusiform gyrus and bilateral amygdala, both of which were reported as brain regions consistently activated for social stimuli (Atzil et al., 2023). In addition, the right medial orbitofrontal cortex (gyrus rectus) was more active in the social condition, which meta-analyses have reported as related to monitoring, learning, and memory of reward reinforcers (Kringelbach, 2005; Liu et al., 2011). The results also confirmed the validity of our social reward stimuli at a neural level.

Some caveats need to be considered while interpreting these results. First, even though the social reward images are real-world images – and offer more ecological validity than point light figures or isolated faces – they are still unrelated to the participant’s current context. The participant does not interact with these stimuli. Future studies should aim to improve further on the ecological validity of social reward conditions by maximizing sensory richness of stimuli and participant engagement (Stijovic et al., 2024). Second, the measure of autistic traits used in this study is a self-report measure, which renders it likely to be influenced by a number of sociological factors including gender, and culture. Future studies could consider moving beyond self-report to index autism-related phenotypic variation in the general population, e.g. through the use of simple behavioral tasks that pertain to different domains of the autistic phenotype (Dubey et al., 2024). Third, larger samples in which there are enough autistic participants would allow analysis of whether patterns are found equally in both diagnosed and non-diagnosed samples, or could highlight if there are qualitative differences in how autistic traits relate to social reward value between these groups. Finally, the limited sample size in the current study did not allow for a meaningful examination of potential sex differences. Future studies with larger, balanced samples should test the impact of sex and its interaction with autistic traits on the reported variables.

In conclusion, we compared the consummatory aspect of social and nonsocial reward processing within a single paradigm in a sample of young adults, using behavioral and fMRI measures. We found that greater functional coupling between the LFG and the ACC was associated with a significantly higher behavioral preference for social over nonsocial rewards. We further found that LFG to ACC connectivity fully mediated the association between autistic traits and the behavioral preference for social over nonsocial rewards. This result points to a potential neural mechanism underlying the observation that individuals with high autistic traits often find social stimuli less rewarding in lab-based tasks, compared to those with low autistic traits. Future work should test the generalisability of this mechanism by probing it further in larger samples and with clinically diagnosed autistic adults and children.

## Supporting information

Haffey et al. supplementary material 1 Haffey et al. supplementary material

Haffey et al. supplementary material 2 Haffey et al. supplementary material

## Data Availability

Data and scripts analyzing it can be accessed at: https://osf.io/jmqv9/

## Supplementary material

The supplementary material for this article can be found at https://doi.org/10.1017/pen.2025.10003.

## Open Practices Statement

Data, material, and analysis code are available by contacting the corresponding author. None of the experiment was preregistered.
