# Supplementary material for: Autistic traits modulate neural and behavioral responses to social vs nonsocial rewards
Source: Personal Neurosci. 2025 Sep 4;8:e4. doi: 10.1017/pen.2025.10003 (PMC12450549; doi:10.1017/pen.2025.10003)
Supplement: Haffey et al. supplementary material 1 — Haffey et al. supplementary material [file S2513988625100035sup001.docx]

**Autistic traits modulate neural and behavioural responses to social vs nonsocial rewards**

Anthony Haffey^1*^, Chun-Ting Hsu^1*^ and Bhismadev Chakrabarti^1,2,3^

1. Centre for Integrative Neuroscience and Neurodynamics,

School of Psychology and Clinical Language Sciences

University of Reading, Whiteknights Campus, Reading RG6 6AL, United Kingdom

2. Department of Psychology, Ashoka University, Sonipat, India

3. India Autism Center, Kolkata, India

* Equally contributed, co-corresponding authors

Correspondence to: Anthony Haffey or Chun Ting-Hsu (contact details below)

Contact information for other authors:

Anthony Haffey, a.haffey@reading.ac.uk

Chun-Ting Hsu, hsuchunting@gmail.com

Keywords: autism, MRI, PPI, connectivity

Data availability statement: Data and scripts analysing it can be accessed at: <https://osf.io/jmqv9/>

**Declarations**

*Funding*

This work was supported by grants from the Medical Research Council UK (G1100359/1) and the Leverhulme Trust (PLP-2015-329) to BC, and an ESRC-MRC interdisciplinary studentship to AH.

*Conflicts of Interest / Competing Interests*

The authors declare they have no financial or non-financial interests.

*Ethics approval*

All procedures performed in studies involving human participants were in accordance with the ethical standards as laid down in the 1964 Declaration of Helsinki and its later amendments or comparable ethical standards. Ethics approval was obtained from the University Research Ethics Committee (Ref. UREC 13/35 ).

*Consent*

All participants gave written informed consent to experiment participation and experimental data publication.

*Author contributions statement*

AH and BC conceptualised the experiment. AH collected the data. BC provided research supervision. AH and C-TH analyzed the results. AH, C-TH and BC co-wrote the manuscript.

*Acknowledgements*

We would like to thank Dr. Garret O’Connell for his help with data collection and our participants for their time.

*Social Media summary*

Smiles vs. objects: fMRI study links social reward preference to specific neural circuits—modulated by autistic traits.

Word count: 5812

## **Abstract**

Social rewards (e.g. smiles) powerfully shape human behaviour, starting from early childhood. Yet, the neural architecture that enables differential processing of social and nonsocial rewards remains largely unknown. Few previous studies that directly compared social vs nonsocial stimuli have used stimuli that have low ecological validity or are not matched on low-level stimulus parameters - limiting the scope of inference. To address this gap in knowledge, social and nonsocial reward images taken from the real world were matched on valence, arousal, and key low-level stimulus properties and presented to 37 adults in an fMRI study. Individual self-reported preference for social images was associated with the functional connectivity between the left anterior insula (LAI) and medial orbitofrontal cortex (mOFC), as well as that between the left Fusiform Gyrus (LFG) and the Anterior Cingulate Cortex (ACC). Autistic traits negatively modulated LAI - mOFC connectivity and LFG – ACC connectivity. Reduced functional connectivity between these regions may contribute to the lower social reward responsivity in individuals with high autistic traits, as also noted from their lower valence ratings to social rewards. This study provides evidence for a new experimental paradigm to test social reward processing at a behavioural and neural level, which can contribute to potential transdiagnostic biomarkers for social cognitive processes.

## **Introduction**

Social rewards such as smiles are crucial components of social interactions, act as powerful reinforcers, and help shape human behaviour and create rapport. Studies in young infants have shown how most children prefer to orient to a face/ voice compared to comparable nonsocial stimuli (Morton & Johnson, 1991; Vouloumanos et al., 2010). Studies in adults have shown a similar preference for social over nonsocial rewards (Chakrabarti et al., 2017; Fletcher-Watson et al., 2008). The preference for social over nonsocial rewards has been tested extensively using behavioural and eye-tracking paradigms (Hedger et al., 2020; Frazier et al., 2018). In contrast, comparatively few studies have directly investigated the neural processing of social vs nonsocial rewards.

The reward system is one of the most-studied networks of brain regions. Insights from electrophysiological studies in rodent and non-human primates as well as from functional MRI studies in humans have revealed the importance of a few key nodes in this network, that include the medial orbitofrontal cortex (mOFC), ventral striatum (VS) and amygdala (Haber & Knutson, 2010). There are fibre tracts between the VS and the mOFC (Haber et al., 2006; Lehéricy et al., 2004). The VS also receives input from the amygdala (Friedman et al., 2002; Fudge et al., 2002; Russchen et al., 1985) in primates, which plays a key role in tagging salience of emotional (Anderson & Phelps, 2001) and rewarding stimuli (Mahler & Berridge, 2009). The majority of studies on the neuroanatomy of reward processing comes from the processing of nonsocial rewards such as food, chocolate, or money.

Studies that have explored social reward processing have found social rewards to activate a similar set of regions to those described earlier (Fareri, Chang, & Delgado, 2012; Fareri, Chang, & Delgado, 2015; Izuma et al., 2008; Lin, Adolphs, et al., 2012; Wake & Izuma, 2017; Zink et al., 2008). The VS, mOFC and amygdala have been shown to respond to social rewards including smiles (Scott-Van Zeeland et al., 2010) positive feedback (Izuma et al., 2008), cooperation (Elliott et al., 2006), social status (Zink et al., 2008), charitable giving (Kuss et al., 2013) and interacting with friends (Güroğlu et al., 2008). In an early study, mOFC activity in response to monetary reward was increased by co-operation to win this money (Elliott et al., 2006), suggesting that this activity could constitute a common neural currency for both social and nonsocial rewards (Grabenhorst et al., 2010). More recently, the temporoparietal junction (TPJ) has been shown to modulate social value processing in the ventromedial prefrontal cortex (Strombach et al. 2015). In addition, the anterior cingulate cortex (ACC) responds to rewards and punishment for conspecifics, supporting its role in social reward processing (Schneider et al., 2020).

Most studies mentioned above presented social rewards in isolation, which do not allow for a direct comparison of social and nonsocial reward processing within the same experimental paradigm (but see Rademacher et al., 2014; Scott-Van Zeeland et al., 2010 for notable exceptions). In recent years, a class of paradigms comparing social vs nonsocial rewards directly involves the use of the monetary incentive delay and the social incentive delay task. (Zhang et al., 2020; Gu et al., 2019). This paradigm requires participants to make and remember associations between shapes/ symbols with reward outcomes and primarily indexes the anticipatory phase of reward processing. In contrast, paradigms directly presenting images of social and nonsocial rewards are likely to primarily index the consummatory phase of reward processing. In the minority of studies that formally compared responses to images of social vs nonsocial rewards, the stimuli either have low ecological validity (e.g. point-light figures) or are not matched for their low-level stimulus properties - which poses challenges for interpretation (Saygin et al., 2004; Sasson et al., 2008).

Differences in individual responsivity to social rewards exist across the population but can be particularly notable in conditions such as autism. Autistic individuals often show reduced responsivity to social rewards in lab-based assessments (Hedger et al., 2020). Lower activity in reward-related brain regions such as mOFC and VS to social rewards have also been reported in autistic compared to non-autistic adults (Kohls, Schulte-Rüther, et al., 2013; Scott-Van Zeeland et al., 2010) but see Dichter et al. (2012) for an exception. A meta-analysis of neuroimaging studies reported differential reward system responses to both social and nonsocial rewards in autistic individuals (Clements et al., 2018). Similar to the studies on non-autistic individuals, the majority of studies included in this meta-analysis examined social or nonsocial reward processing in isolation.

To address the gaps in the literature, the current study aimed to develop and test a new stimuli set and paradigm to investigate neural processing of social vs. nonsocial rewards, when these different categories of stimuli are closely matched on stimulus parameters. The stimuli were drawn from well-characterised real-world images and hence have higher ecological validity than schematic faces/ posed expressions. However, it is worth noting that all the pictures may not evoke strong subjective feelings of reward. To this end, our use of the term 'reward' is more aligned with the definition of emotion proposed by Ralph Adolphs - in proposing that it is a functional state that can enable subjective feelings of pleasure, as well as behavioural changes (Fox et al., 2018). A secondary aim was to investigate if individual differences in functional connectivity between key brain nodes involved in reward and social processing was associated with autistic traits. In light of the previous studies on autism and social rewards, we hypothesised that individuals with high autistic traits will have reduced preference for social rewards, and have reduced functional connectivity between brain regions involved in social reward processing (Chevallier et al., 2012)

## **Materials and Methods**

*Participants*

40 participants completed the study from the local population in and around the university. Two participants were excluded from the analysis due to a programming error and one participant was removed due to excessive movement (see *data analysis*), leaving 37 participants (19 females; mean age = 22.89 years, S.D. = 5.02 years, min = 18, max = 40). All participants had normal or corrected to normal vision and completed the 50 item Autism Spectrum Quotient with binarized response format (AQ; Baron-Cohen et al., 2001). The mean AQ score was 14.3 (SD=7.74), the scores ranged from 3-33 and a higher score reflects more autistic traits. Participants were not asked about whether they had a clinical diagnosis of any neurodevelopmental conditions, such as autism. Autistic traits are continuously distributed between those with and without a diagnosis (Robinson et al., 2011).

*Design and Materials*

Forty pairs of social and nonsocial reward images (images can be requested from the authors) were taken from the International Affective Picture System (Lang et al., 1999) and downloaded from the website Flickr. Social reward images included one or more humans (e.g. happy individuals), while nonsocial reward images included rewarding nonsocial objects (e.g. food and scenery). The social images comprised happy children and adults in different real-world contexts ranging from weddings, sports events, social events, and family photographs. Half of the nonsocial images included food, and the remaining images were of scenery, vehicles, money and one piece of jewellery.

The images were modified with ImageJ (Abràmoff, 2004) and Adobe Photoshop to match them for size and multiple image parameters (e.g. contrast and luminance). The Koch toolbox (Walther & Koch, 2006) was used to calculate image saliency for each pair of images in a previous paper, and there was no mean difference in luminosity, RMS contrast (local and global), as well as Koch toolbox metric of image saliency between social and nonsocial images (Chakrabarti et al., 2017). Previous studies using these stimuli have demonstrated greater gaze fixation for social over nonsocial images using eye-tracking, and quicker subjective awareness of social over nonsocial stimuli using continuous flash suppression (Chakrabarti et al., 2017; Gray et al., 2018; Hedger et al., 2018, 2020).

All stimuli were displayed using E-prime 2.0 (Psychology Software Tools, PA, USA) on a ViewSonic VE510s monitor (ViewSonic Corporation, California, USA).

*Procedures*

Participants performed a rating task outside the scanner and a fMRI task. During the rating task, participants were presented with an image to give a valence rating on a 9-point likert scale using the manikins from (Bradley & Lang, 1994). Participants were instructed to rate how happy the images were by clicking one of 9 manikins conveying a range of emotions from unhappy to happy.

During the fMRI task, the social and nonsocial images were presented in pseudo-randomised blocks of four images of the same condition (social/ nonsocial) followed by a block of four subsequent fixation crosses. The order of presentation of social and nonsocial images was randomised within these constraints. Each image was presented for 4 seconds, fixation crosses for 3 seconds followed by 1 second of blank screen. To maintain attention to the task, participants were required to press a button with their right index finger during each presentation of an image or fixation cross. The 50-item Autism Quotient questionnaire (AQ; Baron-Cohen et al., 2001) was completed online after the scanning session.

*Valence ratings*

Across the whole sample, t-tests were used to test a) whether the images are perceived as rewarding and b) that social images were not significantly different in their reward value to nonsocial images. At an individual level, mean valence ratings were calculated separately for social reward images and nonsocial reward images. Pearson’s r was computed for correlations between AQ and these mean ratings to test whether autistic traits were associated with diminished reward value for social rewards. Additionally, a composite metric for quantifying individual preference for social vs. nonsocial rewards (i.e. ***sociality bias***) was computed by subtracting nonsocial from social mean valence ratings. Valence ratings that were 3 or more SDs away from the mean across both social and nonsocial images were removed as outliers for each participant.

*Regions of interest*

Based on our a priori hypotheses, we selected our specific regions of interest (ROIs) based on meta-analyses of sociality- (Atzil et al., 2023) and reward-related (Liu et al., 2011) brain regions. The masks for sociality-related regions were defined functionally by the activation maps of Atzil et al.’s multilevel peak kernel density analysis on the contrast: [All social stimuli > No social stimuli] (bilateral amygdala, bilateral FG, and right TPJ, Fig. 1A). The masks for reward-related regions were defined functionally by making 10mm spheres centered on coordinates of brain areas activated by reward outcome (bilateral VS: [12, 10 -6] and [-10 8 -4], and one mask made of two overlapping spheres of bilateral mOFC: [-2 56 -6] and [2 48 -14], and ACC [8 24 32], Fig. 1B) reported in Liu et al.

**Figure 1**

*Predefined regions of interest. A. Axial sections at MNI z = -18 (left) and z = 7 (right) showing ROIs defined functionally by the activation maps of Atzil et al.’s multilevel peak kernel density analysis: left amygdala (red), right amygdala (blue), left FG (green), right FG (magenta), and right TPJ (yellow). B. Sections at MNI x = 0 (upper right), y = 9 (upper left), and z = 32 (lower) showing ROIs defined functionally by making 10 mm spheres centered on coordinates of brain areas activated by reward outcome reported in Liu et al.: left VS (red) and right VS (blue)], mOFC (green), and ACC (magenta).*


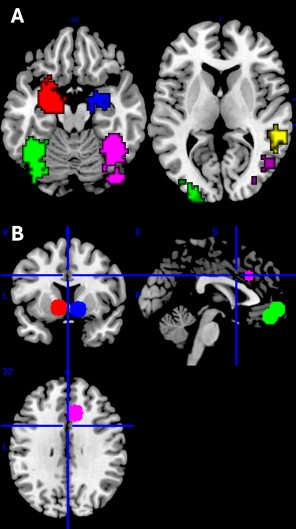


*fMRI data acquisition and preprocessing*

Scans were conducted using a 3T Siemens TIM Trio MRI scanner with 12-channel head coil. One run of 322 volumes was measured per participant using a T_2_*-weighted echo-planar sequence [slice thickness: 3 mm, no gap, 37 slices, repetition time (TR): 2s, echo time (TE): 30ms, echo spacing: 0.53ms, GRAPPA acceleration factor: 2, flip angle: 90°, matrix: 64 × 64, field of view (FOV): 192 mm, voxel size: 3.0 mm × 3.0 mm × 3.0 mm] and individual high-resolution T1- weighted anatomical data (MPRAGE sequence) were acquired (TR: 2.2, TE: 2.9, FOV: 250, matrix: 256 × 256, sagittal plane, slice thickness: 1 mm, 176 slices, resolution: 1.0 mm × 1.0 mm × 1.0 mm). The phase and magnitude images for the construction of the field map were collected with the same positions and dimensions as the functional scans [slice thickness: 3 mm, no gap, 32 slices, TR: 400 ms, TE: 5.19 and 7.65 ms, flip angle: 60°, matrix: 64 × 64, FOV: 192 mm, voxel size: 3.0 mm × 3.0 mm × 3.0 mm].

The data was then further preprocessed and analyzed using MATLAB 2024b and the software package SPM25 (www.fil.ion.ucl.ac.uk/spm). Preprocessing consisted of slice-timing correction, realignment for motion correction, unwarping with the field map in the first and second order for rotations along the x and y axis, and coregistration of the structural image to the mean realigned functional image. Structural images were segmented into grey matter, white matter, cerebrospinal fluid, bone, soft tissue, and air/background with the ‘New Segment’ module (Ashburner & Friston, 2005). A group anatomical template was created with DARTEL (Diffeomorphic Anatomical Registration using Exponentiated Lie algebra; Ashburner, 2007) toolbox from the segmented grey and white matter images. Transformation parameters for structural images were then applied to functional images to normalize them to the brain template of the Montreal Neurological Institute (MNI) supplied with SPM. Functional images were spatially smoothed with a kernel of 6 mm full-width-at-half-maximum after normalization.

*fMRI mass univariate GLM analysis*

Two participants were removed due to programming error: blocks were presented in an entirely random order to the first two participants, resulting in clusters of 5 blocks of the same stimulus type in a row for both. The block order of the remaining participants was pseudo-randomised so that half the participants received one order of social and nonsocial blocks, whereas the other received the reversed order. Due to the difference between the block structure in the first two participants and the remaining 38, these two participants were not included in the analysis. One further participant’s data was removed due to excessive movement, having moved more than 3 mm between two volumes, leaving 37 participants.

Statistical parametric maps were calculated with multiple regressions of the data onto a model of the hemodynamic response (Friston et al., 1995). The first level general linear model analyses contained two psychological regressors of interest for “Social” and “Nonsocial” conditions in the design matrix. Each block in each condition lasted 16 seconds. Psychological regressors were convolved with the canonical hemodynamic response function. Six motion parameters were included as nuisance regressors. A high pass filter with a cut off period of 128 s was applied, and the temporal autocorrelation was accounted for with the AR(1) option. For each ROI, the first eigenvariate of the contrast [social - nonsocial] for each participant were extracted with MarsBaR (version 0.45).

*fMRI mass univariate PPI analysis*

In order to test whether the sociality-and reward-associated brain activity in ROIs is functionally coupled, a generalised Psychophysiological Interaction analysis (gPPI toolbox v13.1; Friston et al., 1997; McLaren et al., 2012) was performed. ROI’s from Atzil’s et al. (2023) were defined as the seed region, while ROIs from Liu et al. (2011) others were defined as target regions. The vector of neural response was estimated by deconvoluting the first eigenvariate of the BOLD signal extracted from the seed region. The interaction vector of each condition was calculated as the product of the estimated neural response vector and the condition vector’s ON times. We then performed the general linear model analysis for the whole brain including the interaction vectors, the condition vectors, and the estimated neural response vector of the seed region as regressors. For each target region, the first eigenvariate of the univariate contrast between the interaction term of the social condition and that of the nonsocial condition for each participant were extracted with MarsBaR (version 0.45) and used for the group level one-sample t-test. To test whether autistic traits modulated the functional connectivity between brain activity associated with social stimuli processing and reward value responsiveness, AQ was correlated with the contrast values of the target regions in the gPPI analysis.

For the purposes of completeness, we provide whole brain analyses in the *supplementary materials.* Family-wise error corrected p < 0.05 at the peak level, cluster size ≧ 5, was used as the significant threshold for whole-brain results.

Participants were identified as correlational outliers if their Cook’s D value was greater than 4/35 (i.e. 4/(N-1-K)) and were removed from the specific correlation that they were outliers for.

All analyses use 1-tailed p-values to reflect the directional nature of the hypotheses that: autistic traits will be associated with reduced sociality bias and reduced connectivity between social and reward regions; sociality bias will be associated with increased connectivity between social and reward regions. Bootstrapped confidence intervals with replacement based on 10,000 repetitions are included for all correlations to address concerns about small sample sizes. Holm–Šidák corrections for multiple testing are applied to correlations between *sociality bias* and connectivity for each pair of regions (see supplementary materials 2).

##

## **Results**

#### *Rating Task*

T-tests of the mean ratings of the images were conducted to test the assumption that the images were similarly pleasant to participants in this sample. No significant difference between social and nonsocial was found across the whole sample (*t*(36) = .47, *p* = .643, 95% CI [-0.3, 0.18], *d* = .08). One sample t-tests found that the mean ratings for social (M=6.47, SD=.69, *t*(36) = 13.01, *p < .*001, 95% CI [6.24, 6.69], *d* = 2.14) and nonsocial images (M=6.52, SD=.63, *t*(36) = 14.66, *p < .*001, 95% CI [6.31, 6.73], *d* = 2.41) were significantly above the neutral rating of 5, suggesting that the participants found the images rewarding. Against expectations, some participants rated some images as unpleasant (less than 5), but this was only true for 5.45% of social images and 4% of nonsocial images across all participants.

AQ correlated negatively with valence ratings of social rewards (*r*(32) = -.49, p = .001; bootstrap CI: [-.68; -.3]) but no such pattern was seen for valence ratings of nonsocial rewards (*r*(33) = -.022, p = .901; bootstrap CI: [-.26; .23]). The negative association between AQ with *sociality bias* was significant (*r*(35) = -.36, *p* = .016; bootstrap CI: [-.54; -.19]), consistent with autistic traits being associated with reduced preference for social than nonsocial images (see Fig. 2).

**Figure 2**

*Scatterplot of associations between autistic traits measured using the Autism Spectrum Quotient (AQ) and social and nonsocial valence ratings.*


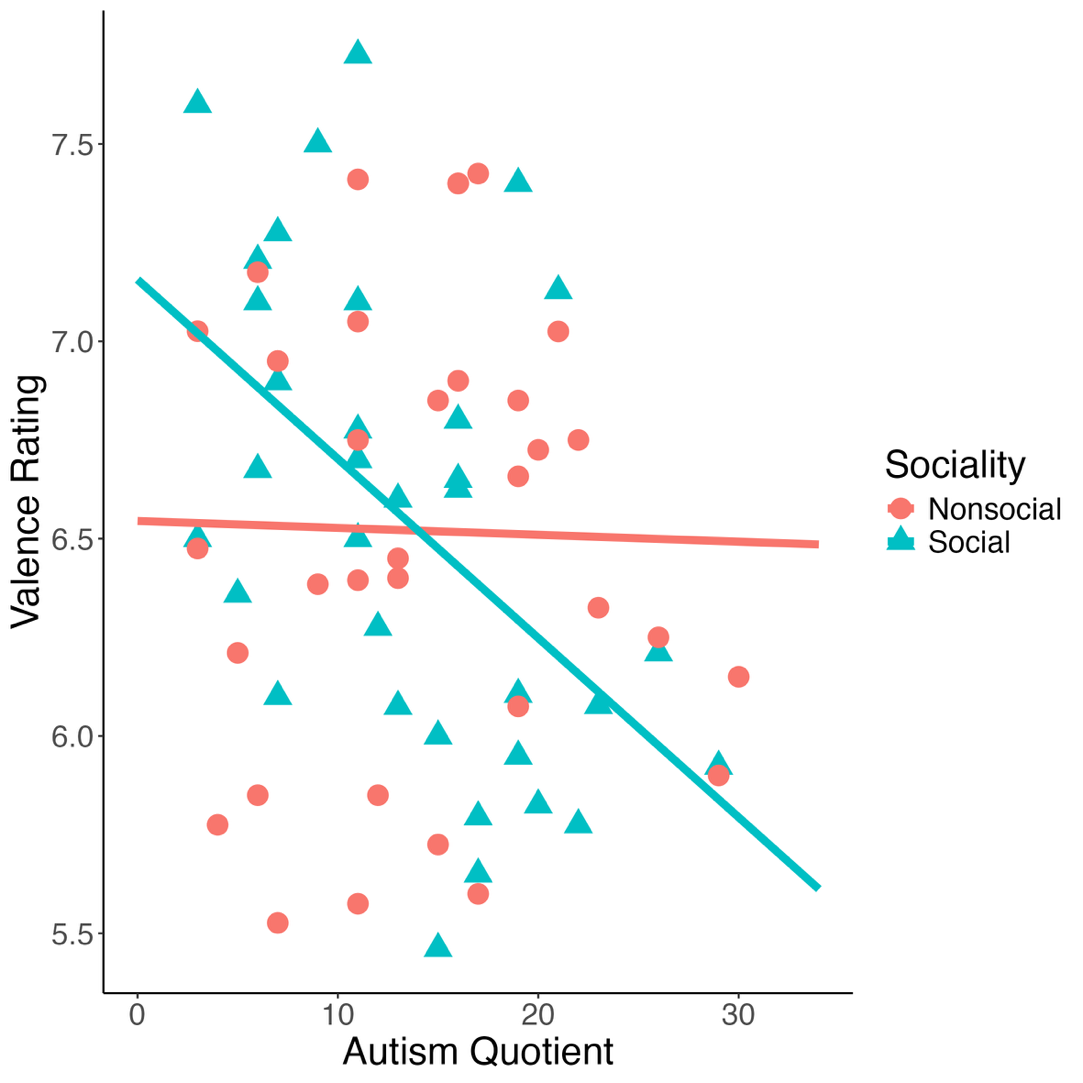


*Note:* These results visualise how autistic traits (AQ) are negatively associated with valence ratings for social (*r*(32) = -.49, *p* = .001) but not nonsocial images (*r*(33) = -.02, *p* = .9)**.**

#### *Brain data*

#### *Whole brain GLM results*

#### At the whole brain level, the following regions showed significantly stronger activity in the social than in the nonsocial condition: bilateral amygdala, right fusiform gyrus, right medial orbitofrontal cortex (gyrus rectus), bilateral temporo-occipital junction. Bilateral fusiform gyrus, visual cortex, and left lateral frontopolar cortex were more active in the nonsocial than in the social condition (Table 1). No significant result was found in the whole-brain correlation of the social-nonsocial contrast values with AQ or social bias.

**Table 1**

*Whole brain results of Social vs. Nonsocial conditions*

| **H** | **Regions** | **Cluster size** | **p (FWE-corr)*** | **T** | **B.A.** | **[x, y, z]** |
| --- | --- | --- | --- | --- | --- | --- |
| **Social > Nonsocial** | | |  |  |  |  |
| R | Inferior occipital | 404 | <.001 | 15.56 | 37 | 51 -72 3 |
|  | Middle temporal |  | <.001 | 12.13 | 37 | 48 -63 12 |
| L | Middle occipital | 285 | <.001 | 11.31 | 19 | -45 -75 9 |
|  | Middle occipital |  | <.001 | 10.97 | 19 | -51 -78 0 |
| R | Fusiform | 53 | <.001 | 9.63 | 37 | 39 -48 -18 |
| R | Amygdala | 13 | <.001 | 7.31 | - | 18 -6 -15 |
| R | Superior temporal | 18 | 0.001 | 7.17 | 21 | 51 -9 -15 |
| R | Gyrus rectus | 11 | 0.001 | 7.16 | 11 | 3 54 -21 |
| R | Temporal pole | 10 | 0.001 | 7.07 | 20 | 42 12 -36 |
| L | Amygdala | 3 | 0.004 | 6.49 | - | -18 -6 -15 |
| **Nonsocial > Social** | | |  |  |  |  |
| L | Fusiform | 88 | <.001 | 11.37 | 37 | -27 -48 -18 |
| L | Middle occipital | 61 | <.001 | 8.12 | 19 | -30 -87 12 |
| R | Fusiform | 59 | <.001 | 8.00 | 37 | 27 -48 -15 |
| R | Middle occipital | 27 | 0.001 | 7.11 | 18 | 30 -81 9 |
| L | Anterior orbital gyrus | 9 | 0.001 | 7.05 | 11 | -24 39 -15 |
| *Note:* Abbreviations: H = hemisphere; L = left; R = right; p = p-value; T = T-value; B.A. = Brodmann area; x, y, z = MNI coordinates* Voxel level FWE-corrected for the whole brain | | | | | | |

####

#### *Brain-Behaviour relationship*

After applying Holm–Šidák corrections to alpha thresholds to address multiple testing, *sociality bias* was positively associated with functional connectivity from the LFG to ACC (r(30) = .64, p < .001; bootstrap CI: [.36; .78]) and the LAI to mOFC (r(34) = .52, p < .001; bootstrap CI: [.35; .66]). AQ was significantly associated with both LFG to ACC (r(31) = -.3, p = .04; bootstrap CI: [-.54; -.05]) and LAI to mOFC connectivity (r(32) = -.45, p = .004; bootstrap CI: [-.67; -.15]).

Mediation analysis found that LFG to ACC connectivity fully mediated the association between AQ and *sociality bias* as the indirect effect was significant (*b* = - .015, , *p* = .044, 95% bootstrapped confidence interval lower limit/LL = -.035, 95% bootstrapped confidence interval upper limit/UL < -.001) but the direct effect was not significant (*b* = -.005, , *p* = .689, bootstrapped LL = -.035, UL = .019; See Fig.3). Mediation analysis found that LAI to mOFC connectivity fully mediated the association between AQ and *sociality bias* as the indirect effect was significant (*b* = - .022, *p* = .031, bootstrapped LL = -.049, UL = -.002) but the direct effect was not significant (*b* = -.01, *p* = .551, LL = -.056, UL = .022; See Fig.4).

**Figure 3**

*Grid of scatterplots summarising the associations between AQ, LFG to ACC connectivity and sociality bias (in blue), with the indirect and direct from mediation analysis (in red).*


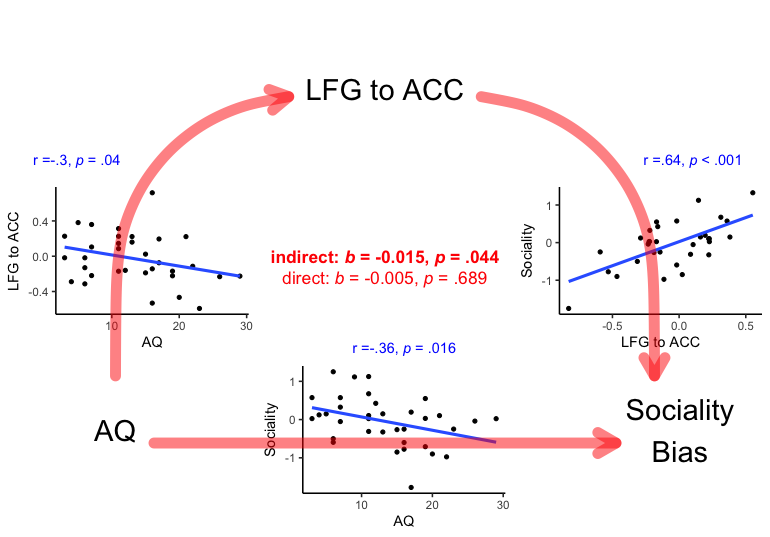


*Note:* all correlations are significant under non-parametric Spearman’s rank correlation (see supplementary analysis 1).

**Figure 4**

*Grid of scatterplots summarising the associations between AQ, LAI to mOFC connectivity and sociality bias (in blue), with the indirect and direct from mediation analysis (in red).*


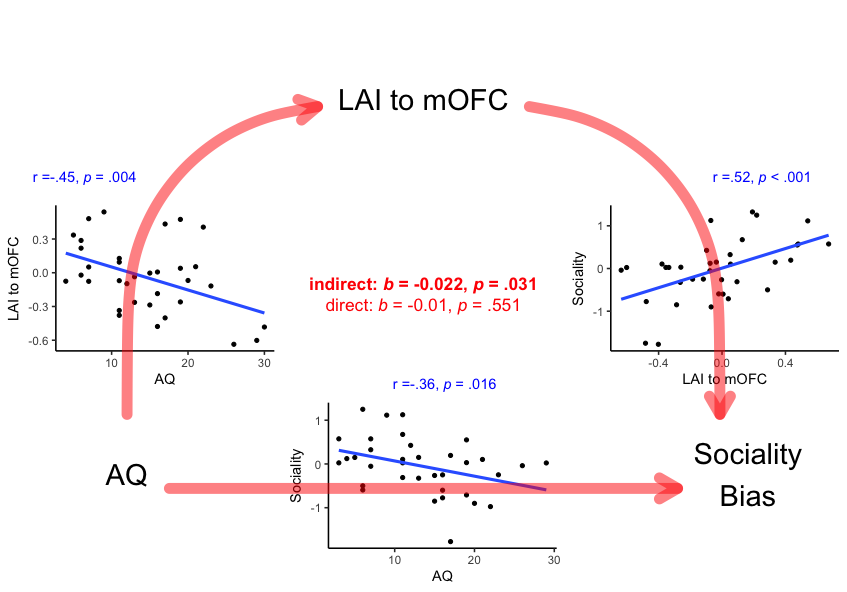


*Note:* all correlations are significant under non-parametric Spearman’s rank correlation (see supplementary analysis 1).

## **Discussion**

We compared response to social vs nonsocial rewards in a sample of young adults using self-report and fMRI. We found that behavioural preference for social over nonsocial rewards (i.e. sociality bias) was positively associated with functional connectivity of LFG-ACC and LAI-mOFC. We further found that the relationship between autistic traits and sociality bias is fully mediated by the functional connectivity of LFG-ACC and LAI-mOFC.

A large number of behavioural and eye-tracking studies have previously compared processing of social vs nonsocial stimuli (Hedger et al., 2020; Frazier et al., 2017). Most of these studies have been conducted in young children, and included both rewarding and neutral stimuli. The few studies that have directly compared social and nonsocial reward processing within a single paradigm have tested the anticipatory component of reward processing rather than the consummatory component of it (Ait Oumeziane et al., 2017; Lin, Adolphs, et al., 2012; Lin, Rangel, et al., 2012; Spreckelmeyer et al., 2009; Wake & Izuma, 2017; although see Rademacher et al., 2010). The current study focuses on the consummatory component of reward processing, by presenting the social and nonsocial reward stimuli to the participants without them having to perform any action. The current design thus allows us to infer the differential neural response to receipt of these different types of reward, without the impact of trade-offs between effort minimization and value maximisation. Additionally, the close matching of the social and nonsocial reward stimuli allows us to make inferences that are less subject to confounds due to stimulus features such as contrast, luminosity, and saliency.

We found that even when social reward images are matched on basic stimulus properties, and rated as equally rewarding to nonsocial reward images across the full sample, autistic traits were coupled with individual preference for social rewards both behaviourally and neurally. At a behavioural level, autistic traits were inversely proportional to the valence ratings for social reward images. This relationship was not seen for nonsocial rewards. This observation is consistent with earlier empirical reports showing a reduced rating of social stimuli or situations by autistic individuals in lab-based studies (Chevallier et al., 2012; Sasson et al., 2012). At the neural level, we found that the functional coupling of LFG-ACC and LAI-mOFC was weaker in individuals with higher autistic traits. While the role of the fusiform gyrus in conspecific face and body processing is well established, a recent study has also identified it as a hub of expression for genes involved in synaptic transmission as well as autism (Chen et al., 2022). Neurochemically, an earlier study reported reduced GABA_B_ receptors in both fusiform gyrus as well as the ACC - which are involved both in maintaining synapses and the excitation-inhibition balance in the brain (Oblak et al., 2010). Functionally, a weaker coupling between fusiform gyrus and anterior cingulate cortex corresponds to reduced links between social perceptual encoding and value encoding. Assuming a certain degree of functional specialisation in the brain, the fusiform gyrus and nearby areas are more involved in perceptual encoding of stimuli (Naspi et al., 2021) - while the ACC is arguably involved more in encoding stimulus value (Cai et al., 2012). This observation is also consistent with earlier accounts of reduced links between reward and social processing in autism (Neufeld et al., 2019; Sims et al., 2014).

The LAI-mOFC functional coupling represented another link between social perceptual and value encoding, which we found to be negatively modulated by autistic traits and further modulate social bias. Anterior insula receives interoceptive and nociceptive afferent input from inner organs and skin (Craig, 2009), and has long been associated with pain empathy (Lamm et al., 2007, 2009, Bernhardt and Singer, 2012) and social norm compliance (Bellucci et al., 2018). Previous studies have investigated the role of interoception in social cognition (Arnold et al., 2019). For example, higher interoceptive awareness is associated with less negative affect after challenging social situations, due to the ability to properly attribute the physiological response as resulting from the external social situation, resulting in better emotional regulation in social situations (Werner et al., 2013). The mOFC is associated with reward value encoding (Kringelbach, 2005; Rolls et al., 2020). In mammals, there is direct projection from anterior insula to orbitofrontal cortex (Mathiasen et al., 2023). In humans the coactivation of LAI and mOFC has been associated with the pro-social behavior of self-impression/reputation management when being observed by others (Yoon et al., 2021), which was said to be a behavioral manifestation of allostatic regulatory process, to prevent homeostatic disturbances accompanied by social threat/stress/punishment (Slavich et al., 2010). In the present study, participants showing preference for social rewards had enhanced functional coupling when viewing social stimuli with human figures, which might be associated with stronger incentive or preparedness for self-impression/reputation management and the consequential social rewards (Tennie et al., 2010).

Interestingly, the functional coupling of LFG-ACC and LAI-mOFC fully mediated the association between AQ and the behavioural preference for social over nonsocial rewards (i.e.*sociality bias*)*.* While autism has been associated with reduced preference for social rewards in several prior studies (Frazier et al., 2017; and specifically using these stimuli by Hedger et al., 2018), direct investigations of underlying mechanisms linking neural and behavioural data have been rare. To this end, the mediation analysis suggests an indicative neural mechanism to explain the behavioural observation of reduced preference for social rewards in individuals with high autistic traits. *However,* similar to correlations, claims about causality from mediation in cross-sectional data are more vulnerable to overlooking causal covariates or confounds than experimental or longitudinal designs (Rohrer et al., 2023).

Finally, we discuss the results of the whole brain univariate contrast for the sake of completeness. These analyses (Table 1) showed that social condition more specifically activated the right fusiform gyrus and bilateral amygdala, both of which were reported as brain regions consistently activated for social stimuli (Atzil et al., 2023). In addition, the right medial orbitofrontal cortex (gyrus rectus) was more active in the social condition, which meta-analyses have reported as related to monitoring, learning and memory of reward reinforcers (Kringelbach, 2005; Liu et al., 2011). The results also confirmed the validity of our social reward stimuli at a neural level.

Some caveats need to be considered while interpreting these results. First, even though the social reward images are real world images - and offer more ecological validity than point light figures or isolated faces - they are still unrelated to the participant’s current context. The participant does not interact with these stimuli. Future studies should aim to improve further on the ecological validity of social reward conditions by maximising sensory richness of stimuli and participant engagement (Stijovic et al., 2024). Second, the measure of autistic traits used in this study is a self-report measure, which renders it likely to be influenced by a number of sociological factors including gender, and culture. Future studies could consider moving beyond self-report to index autism-related phenotypic variation in the general population, e.g. through the use of simple behavioural tasks that pertain to different domains of the autistic phenotype (Dubey et al., 2023). Third, larger samples in which there are enough autistic participants would allow analysis of whether patterns are found equally in both diagnosed and non-diagnosed samples, or could highlight if there are qualitative differences in how autistic traits relate to social reward value between these groups. Finally, the limited sample size in the current study did not allow for a meaningful examination of potential sex differences. Future studies with larger, balanced samples should test the impact of sex and its interaction with autistic traits on the reported variables.

In conclusion, we compared the consummatory aspect of social and nonsocial reward processing within a single paradigm in a sample of young adults, using behavioural and fMRI measures. We found that greater functional coupling between the LFG and the ACC was associated with a significantly higher behavioural preference for social over nonsocial rewards. We further found that LFG to ACC connectivity fully mediated the association between autistic traits and the behavioural preference for social over nonsocial rewards. This result points to a potential neural mechanism underlying the observation that individuals with high autistic traits often find social stimuli less rewarding in lab-based tasks, compared to those with low autistic traits. Future work should test the generalisability of this mechanism by probing it further in larger samples and with clinically diagnosed autistic adults and children.

**Declarations**

**Funding**

This work was supported by grants from the Medical Research Council UK (G1100359/1) and the Leverhulme Trust (PLP-2015-329) to BC, and an ESRC-MRC interdisciplinary studentship to AH.

**Conflicts of Interest / Competing Interests**

The authors declare they have no financial or non-financial interests.

**Ethics approval**

All procedures performed in studies involving human participants were in accordance with the ethical standards as laid down in the 1964 Declaration of Helsinki and its later amendments or comparable ethical standards. Ethics approval was obtained from the University Research Ethics Committee (Ref. UREC 13/35 ).

**Consent**

All participants gave written informed consent to experiment participation and experimental data publication.

**Author contributions statement**

AH and BC conceptualised the experiment. AH collected the data. BC provided research supervision. AH and C-TH analyzed the results. AH, C-TH and BC co-wrote the manuscript.

**Open Practices Statement**

Data, material, and analysis code is available by contacting the corresponding author. None of the experiment was preregistered.

**References**

Abràmoff, D. M. D. (2004). Image Processing with ImageJ. *Biophotonics International*, *11*, 36–41.

Adamson, K., & Troiani, V. (2018). Distinct and overlapping fusiform activation to faces and food. *NeuroImage*, *174*, 393–406. https://doi.org/10.1016/j.neuroimage.2018.02.064

Ait Oumeziane, B., Schryer-Praga, J., & Foti, D. (2017). “Why don’t they ‘like’ me more?”: Comparing the time courses of social and monetary reward processing. *Neuropsychologia*, *107*, 48–59. https://doi.org/10.1016/j.neuropsychologia.2017.11.001

Anderson, A. K., & Phelps, E. A. (2001). Lesions of the human amygdala impair enhanced perception of emotionally salient events. *Nature*, *411*, 305–309. <https://doi.org/10.1038/35077083>

Arnold, A. J., Winkielman, P., & Dobkins, K. (2019). *Interoception and Social Connection. Frontiers in Psychology*, 10, 2589.<https://doi.org/10.3389/fpsyg.2019.02589>

Ashburner, J. (2007). A fast diffeomorphic image registration algorithm. *NeuroImage*, *38*, 95–113. https://doi.org/10.1016/j.neuroimage.2007.07.007

Ashburner, J., & Friston, K. J. (2005). Unified segmentation. *NeuroImage*, *26*, 839–851. <https://doi.org/10.1016/j.neuroimage.2005.02.018>

Atzil, S., Satpute, A. B., Zhang, J., Parrish, M. H., Shablack, H., MacCormack, J. K., ... & Lindquist, K. A. (2023). The impact of sociality and affective valence on brain activation: a meta-analysis. *NeuroImage*, *268*, 119879.

Baron-Cohen, S., & Wheelwright, S. (2004). The empathy quotient: An investigation of adults with Asperger syndrome or high functioning autism, and normal sex differences. *Journal of Autism and Developmental Disorders*, *34*, 163–175. https://doi.org/10.1023/B:Jadd.0000022607.19833.00

Baron-Cohen, S., Wheelwright, S., Skinner, R., Martin, J., & Clubley, E. (2001). The autism-spectrum quotient (AQ): Evidence from Asperger syndrome/high-functioning autism, males and females, scientists and mathematicians. *Journal of Autism and Developmental Disorders*, *31*, 5–17.

Beauchamp, M. S., Lee, K. E., Haxby, J. V., & Martin, A. (2002). Parallel Visual Motion Processing Streams for Manipulable Objects and Human Movements. *Neuron*, *34*, 149–159. <https://doi.org/10.1016/S0896-6273(02)00642-6>

Bellucci, G., Feng, C., Camilleri, J., Eickhoff, S. B., & Krueger, F. (2018). The role of the anterior insula in social norm compliance and enforcement: Evidence from coordinate-based and functional connectivity meta-analyses. *Neuroscience & Biobehavioral Reviews*, 92, 378–389.<https://doi.org/10.1016/j.neubiorev.2018.06.024>

Bernhardt, B. C., & Singer, T. (2012). The Neural Basis of Empathy. *Annual Review of Neuroscience*, 35, 1–23.<https://doi.org/10.1146/annurev-neuro-062111-150536>

Bottema-Beutel, K., Kim, S. Y., & Crowley, S. (2019). A systematic review and meta-regression analysis of social functioning correlates in autism and typical development: Bottema-Beutel et al./Social functioning meta-analysis. *Autism Research*, *12*, 152–175. https://doi.org/10.1002/aur.2055

Bradley, M. M., & Lang, P. J. (1994). Measuring emotion: The self-assessment manikin and the semantic differential. *Journal of Behavior Therapy and Experimental Psychiatry*, *25*, 49–59. <https://doi.org/10.1016/0005-7916(94)90063-9>

Cai, X., & Padoa-Schioppa, C. (2012). Neuronal encoding of subjective value in dorsal and ventral anterior cingulate cortex. *Journal of Neuroscience*, *32*, 3791-3808. https://doi.org/10.1523/JNEUROSCI.0677-21.2021

Chakrabarti, B., Bullmore, E. T., & Baron-Cohen, S. (2006). Empathizing with basic emotions: Common and discrete neural substrates. *Social Neuroscience*, *1*, 364–384. https://doi.org/Doi 10.1080/17470910601041317

Chakrabarti, B., Haffey, A., Canzano, L., Taylor, C. P., & McSorley, E. (2017). Individual differences in responsivity to social rewards: Insights from two eye-tracking tasks. *PLOS ONE*, *12*, e0185146. <https://doi.org/10.1371/journal.pone.0185146>

Chen, J., Zhang, C., Wang, R., Jiang, P., Cai, H., Zhao, W., ... & Yu, Y. (2022). Molecular basis underlying functional connectivity of fusiform gyrus subregions: A transcriptome-neuroimaging spatial correlation study. *Cortex*, *152*, 59-73. https://doi.org/10.1016/j.cortex.2022.03.016

Chevallier, C., Kohls, G., Troiani, V., Brodkin, E. S., & Schultz, R. T. (2012). The social motivation theory of autism. *Trends in Cognitive Sciences*, *16*, 231–239. https://doi.org/10.1016/j.tics.2012.02.007

Clements, C. C., Zoltowski, A. R., Yankowitz, L. D., Yerys, B. E., Schultz, R. T., & Herrington, J. D. (2018). Evaluation of the Social Motivation Hypothesis of Autism: A Systematic Review and Meta-analysis. *JAMA Psychiatry*, *75*, 797–808. <https://doi.org/10.1001/jamapsychiatry.2018.1100>

Craig, A. D. (2009). How do you feel—Now? The anterior insula and human awareness. *Nature Reviews Neuroscience*, 10, 59–70.<https://doi.org/10.1038/nrn2555>

Desikan, R. S., Segonne, F., Fischl, B., Quinn, B. T., Dickerson, B. C., Blacker, D., Buckner, R. L., Dale, A. M., Maguire, R. P., Hyman, B. T., Albert, M. S., & Killiany, R. J. (2006). An automated labeling system for subdividing the human cerebral cortex on MRI scans into gyral based regions of interest. *Neuroimage*, *31*, 968–980. https://doi.org/DOI 10.1016/j.neuroimage.2006.01.021

Dichter, G. S., Felder, J. N., Green, S. R., Rittenberg, A. M., Sasson, N. J., & Bodfish, J. W. (2012). Reward circuitry function in autism spectrum disorders. *Social Cognitive and Affective Neuroscience*, *7*, 160–172. <https://doi.org/10.1093/scan/nsq095>

Dubey, I., Bishain, R., Dasgupta, J., Bhavnani, S., Belmonte, M. K., Gliga, T., ... & Chakrabarti, B. (2024). Using mobile health technology to assess childhood autism in low-resource community settings in India: An innovation to address the detection gap. *Autism*, *28*, 755-769. https://doi.org/10.1177/13623613231182801

Elliott, R., Völlm, B., Drury, A., McKie, S., Richardson, P., & William Deakin, J. F. (2006). Co-operation with another player in a financially rewarded guessing game activates regions implicated in theory of mind. *Social Neuroscience*, *1*, 385–395. https://doi.org/10.1080/17470910601041358

Fareri, D. S., Chang, L. J., & Delgado, M. R. (2015). Computational substrates of social value in interpersonal collaboration. Journal of Neuroscience, 35, 8170-8180. https://doi.org/10.1523/JNEUROSCI.4775-14.2015

Fareri, D. S., Chang, L. J., & Delgado, M. R. (2012). Effects of direct social experience on trust decisions and neural reward circuitry. Frontiers in neuroscience, 6, 148.

Fletcher-Watson, S., Findlay, J. M., Leekam, S. R., & Benson, V. (2008). Rapid Detection of Person Information in a Naturalistic Scene. *Perception*, *37*, 571–583. <https://doi.org/10.1068/p5705>

Fox, A. S., Lapate, R. C., Shackman, A. J., & Davidson, R. J. (Eds.). (2018). *The nature of emotion: Fundamental questions*. Oxford University Press.

Frazier, T. W., Strauss, M., Klingemier, E. W., Zetzer, E. E., Hardan, A. Y., Eng, C., & Youngstrom, E. A. (2017). A meta-analysis of gaze differences to social and nonsocial information between individuals with and without autism. *Journal of the American Academy of Child & Adolescent Psychiatry*, *56*, 546-555. https://doi.org/10.1016/j.jaac.2017.05.005

Friedman, D. P., Aggleton, J. P., & Saunders, R. C. (2002). Comparison of hippocampal, amygdala, and perirhinal projections to the nucleus accumbens: Combined anterograde and retrograde tracing study in the Macaque brain. *The Journal of Comparative Neurology*, *450*, 345–365. https://doi.org/10.1002/cne.10336

Friston, K. J., Buechel, C., Fink, G. R., Morris, J., Rolls, E., & Dolan, R. J. (1997). Psychophysiological and modulatory interactions in neuroimaging. *NeuroImage*, *6*, 218–229. https://doi.org/10.1006/nimg.1997.0291

Friston, K. J., Holmes, A. P., Poline, J. B., Grasby, P. J., Williams, S. C., Frackowiak, R. S., & Turner, R. (1995). Analysis of fMRI time-series revisited. *NeuroImage*, *2*, 45–53. https://doi.org/10.1006/nimg.1995.1007

Fudge, J. L., Kunishio, K., Walsh, P., Richard, C., & Haber, S. N. (2002). Amygdaloid projections to ventromedial striatal subterritories in the primate. *Neuroscience*, *110*, 257–275. https://doi.org/10.1016/S0306-4522(01)00546-2

Fusar-Poli, P., Placentino, A., Carletti, F., Landi, P., Allen, P., Surguladze, S., Benedetti, F., Abbamonte, M., Gasparotti, R., Barale, F., Perez, J., McGuire, P., & Politi, P. (2009). Functional atlas of emotional faces processing: A voxel-based meta-analysis of 105 functional magnetic resonance imaging studies. *Journal of Psychiatry & Neuroscience*, *34*, 418–432.

Gossen, A., Groppe, S. E., Winkler, L., Kohls, G., Herrington, J., Schultz, R. T., Grunder, G., & Spreckelmeyer, K. N. (2014). Neural evidence for an association between social proficiency and sensitivity to social reward. *Social Cognitive and Affective Neuroscience*, *9*, 661–670. https://doi.org/10.1093/scan/nst033

Grabenhorst, F., D’Souza, A. A., Parris, B. A., Rolls, E. T., & Passingham, R. E. (2010). A common neural scale for the subjective pleasantness of different primary rewards. *NeuroImage*, *51*, 1265–1274. https://doi.org/10.1016/j.neuroimage.2010.03.043

Gray, K. L. H., Haffey, A., Mihaylova, H. L., & Chakrabarti, B. (2018). Lack of Privileged Access to Awareness for Rewarding Social Scenes in Autism Spectrum Disorder. *Journal of Autism and Developmental Disorders*, *48*, 3311–3318.<https://doi.org/10.1007/s10803-018-3595-9>

Gu, R., Huang, W., Camilleri, J., Xu, P., Wei, P., Eickhoff, S. B., & Feng, C. (2019). Love is analogous to money in human brain: Coordinate-based and functional connectivity meta-analyses of social and monetary reward anticipation. *Neuroscience & Biobehavioral Reviews*, 100, 108-128.

Güroğlu, B., Haselager, G. J. T., van Lieshout, C. F. M., Takashima, A., Rijpkema, M., & Fernández, G. (2008). Why are friends special? Implementing a social interaction simulation task to probe the neural correlates of friendship. *NeuroImage*, *39*, 903–910. https://doi.org/10.1016/j.neuroimage.2007.09.007

Haber, S. N., Kim, K.-S., Mailly, P., & Calzavara, R. (2006). Reward-Related Cortical Inputs Define a Large Striatal Region in Primates That Interface with Associative Cortical Connections, Providing a Substrate for Incentive-Based Learning. *Journal of Neuroscience*, *26*, 8368–8376. https://doi.org/10.1523/JNEUROSCI.0271-06.2006

Haber, S. N., & Knutson, B. (2010). The reward circuit: Linking primate anatomy and human imaging. *Neuropsychopharmacology*, *35*, 4–26. https://doi.org/10.1038/npp.2009.129

Haxby, J. V., Gobbini, M. I., Furey, M. L., Ishai, A., Schouten, J. L., & Pietrini, P. (2001). Distributed and overlapping representations of faces and objects in ventral temporal cortex. *Science*, *293*, 2425–2430. https://doi.org/10.1126/science.1063736

Hebart, M. N., Gorgen, K., & Haynes, J. D. (2014). The Decoding Toolbox (TDT): A versatile software package for multivariate analyses of functional imaging data. *Front Neuroinform*, *8*, 88. https://doi.org/10.3389/fninf.2014.00088

Hedger, N., Dubey, I., & Chakrabarti, B. (2020). Social orienting and social seeking behaviors in ASD. A meta analytic investigation. *Neuroscience & Biobehavioral Reviews*, *119*, 376–395. https://doi.org/10.1016/j.neubiorev.2020.10.003

Hedger, N., Haffey, A., McSorley, E., & Chakrabarti, B. (2018). Empathy modulates the temporal structure of social attention. *Proceedings of the Royal Society B: Biological Sciences*, *285*, 20181716. https://doi.org/10.1098/rspb.2018.1716

Izuma, K., Saito, D. N., & Sadato, N. (2008). Processing of social and monetary rewards in the human striatum. *Neuron*, *58*, 284–294. https://doi.org/10.1016/j.neuron.2008.03.020

Kohls, G., Perino, M. T., Taylor, J. M., Madva, E. N., Cayless, S. J., Troiani, V., Price, E., Faja, S., Herrington, J. D., & Schultz, R. T. (2013). The nucleus accumbens is involved in both the pursuit of social reward and the avoidance of social punishment. *Neuropsychologia*, *51*, 2062–2069. https://doi.org/10.1016/j.neuropsychologia.2013.07.020

Kohls, G., Schulte-Rüther, M., Nehrkorn, B., Müller, K., Fink, G. R., Kamp-Becker, I., Herpertz-Dahlmann, B., Schultz, R. T., & Konrad, K. (2013). Reward system dysfunction in autism spectrum disorders. *Social Cognitive and Affective Neuroscience*, *8*, 565–572. <https://doi.org/10.1093/scan/nss033>

Kringelbach, M. L. (2005). The human orbitofrontal cortex: Linking reward to hedonic experience. *Nat Rev Neurosci*, 6, 691–702.<https://doi.org/10.1038/nrn1747>

Kuss, K., Falk, A., Trautner, P., Elger, C. E., Weber, B., & Fliessbach, K. (2013). A reward prediction error for charitable donations reveals outcome orientation of donators. *Social Cognitive and Affective Neuroscience*, *8*, 216–223. <https://doi.org/10.1093/scan/nsr088>

Lamm, C., Batson, C. D., & Decety, J. (2007). The Neural Substrate of Human Empathy: Effects of Perspective-taking and Cognitive Appraisal. *Journal of Cognitive Neuroscience*, 19, 42–58.<https://doi.org/10.1162/jocn.2007.19.1.42>

Lamm, C., Decety, J., & Singer, T. (2011). Meta-analytic evidence for common and distinct neural networks associated with directly experienced pain and empathy for pain. *NeuroImage*, 54, 2492–2502.<https://doi.org/10.1016/j.neuroimage.2010.10.014>

Lang, P. J., Bradley, M. M., & Cuthbert, B. N. (1999). *International affective picture system (IAPS): Technical manual and affective ratings*. http://www.hsp.epm.br/dpsicobio/Nova_versao_pagina_psicobio/adap/instructions.pdf

Lehéricy, S., Ducros, M., Van De Moortele, P.-F., Francois, C., Thivard, L., Poupon, C., Swindale, N., Ugurbil, K., & Kim, D.-S. (2004). Diffusion tensor fiber tracking shows distinct corticostriatal circuits in humans: DTI Corticostriatal Fibers. *Annals of Neurology*, *55*, 522–529. https://doi.org/10.1002/ana.20030

Lin, A., Adolphs, R., & Rangel, A. (2012). Social and monetary reward learning engage overlapping neural substrates. *Social Cognitive and Affective Neuroscience*, *7*, 274–281. https://doi.org/10.1093/scan/nsr006

Lin, A., Rangel, A., & Adolphs, R. (2012). Impaired Learning of Social Compared to Monetary Rewards in Autism. *Frontiers in Neuroscience*, *6*. https://doi.org/10.3389/fnins.2012.00143

Mahler, S. V., & Berridge, K. C. (2009). Which Cue to “Want?” Central Amygdala Opioid Activation Enhances and Focuses Incentive Salience on a Prepotent Reward Cue. *Journal of Neuroscience*, *29*, 6500–6513.<https://doi.org/10.1523/JNEUROSCI.3875-08.2009>

Martins, D., Rademacher, L., Gabay, A. S., Taylor, R., Richey, J. A., Smith, D. V., ... & Paloyelis, Y. (2021). Mapping social reward and punishment processing in the human brain: a voxel-based meta-analysis of neuroimaging findings using the social incentive delay task. *Neuroscience & Biobehavioral Reviews*, 122, 1-17.

Mathiasen, M. L., Aggleton, J. P., & Witter, M. P. (2023). Projections of the insular cortex to orbitofrontal and medial prefrontal cortex: A tracing study in the rat. Frontiers in Neuroanatomy, 17, 1131167.<https://doi.org/10.3389/fnana.2023.1131167>

McLaren, D. G., Ries, M. L., Xu, G., & Johnson, S. C. (2012). A generalized form of context-dependent psychophysiological interactions (gPPI): A comparison to standard approaches. *NeuroImage*, *61*, 1277–1286. https://doi.org/10.1016/j.neuroimage.2012.03.068

Morton, J., & Johnson, M. H. (n.d.). *CONSPEC and CONLERN: A Two-Process Theory of Infant Face Recognition*. 18.

Müller, R.-A., & Fishman, I. (2018). Brain Connectivity and Neuroimaging of Social Networks in Autism. *Trends in Cognitive Sciences*, *22*, 1103–1116. https://doi.org/10.1016/j.tics.2018.09.008

Naspi, L., Hoffman, P., Devereux, B., & Morcom, A. M. (2021). Perceptual and semantic representations at encoding contribute to true and false recognition of objects. *Journal of Neuroscience*, *41*, 8375-8389.

Neufeld, J., Hsu, C.-T., & Chakrabarti, B. (2019). Atypical Reward-Driven Modulation of Mimicry-Related Neural Activity in Autism. *Frontiers in Psychiatry*, *10*. https://doi.org/10.3389/fpsyt.2019.00327

Oblak, A. L., Gibbs, T. T., & Blatt, G. J. (2010). Decreased GABA B receptors in the cingulate cortex and fusiform gyrus in Autism. Journal of Neurochemistry, 114, 1414–1423. https://doi.org/10.1111/j.1471-4159.2010.06858.x

O’Neill, P.-K., Gore, F., & Salzman, C. D. (2018). Basolateral amygdala circuitry in positive and negative valence. *Current Opinion in Neurobiology*, *49*, 175–183. https://doi.org/10.1016/j.conb.2018.02.012

Pryce, C. R. (2018). Comparative evidence for the importance of the amygdala in regulating reward salience. *Current Opinion in Behavioral Sciences*, *22*, 76–81. https://doi.org/10.1016/j.cobeha.2018.01.023

Rademacher, L., Krach, S., Kohls, G., Irmak, A., Gründer, G., & Spreckelmeyer, K. N. (2010). Dissociation of neural networks for anticipation and consumption of monetary and social rewards. *NeuroImage*, *49*, 3276–3285. https://doi.org/10.1016/j.neuroimage.2009.10.089

Rademacher, L., Salama, A., Gründer, G., & Spreckelmeyer, K. N. (2014). Differential patterns of nucleus accumbens activation during anticipation of monetary and social reward in young and older adults. *Social Cognitive and Affective Neuroscience*, *9*, 825–831.<https://doi.org/10.1093/scan/nst047>

Robinson, E. B., Koenen, K. C., McCormick, M. C., Munir, K., Hallett, V., Happé, F., Plomin, R., & Ronald, A. (2011). Evidence that autistic traits show the same etiology in the general population and at the quantitative extremes (5%, 2.5%, and 1%). *Archives of general psychiatry*, *68*, 1113-1121.

Rolls, E. T., Cheng, W., & Feng, J. (2020). The orbitofrontal cortex: Reward, emotion and depression. Brain Communications, 2, fcaa196.<https://doi.org/10.1093/braincomms/fcaa196>

Rosen, N. E., Lord, C., & Volkmar, F. R. (2021). The Diagnosis of Autism: From Kanner to DSM-III to DSM-5 and Beyond. *Journal of Autism and Developmental Disorders*. https://doi.org/10.1007/s10803-021-04904-1

Russchen, F. T., Bakst, I., Amaral, D. G., & Price, J. L. (1985). The amygdalostriatal projections in the monkey. An anterograde tracing study. *Brain Research*, *329*, 241–257. https://doi.org/10.1016/0006-8993(85)90530-X

Sasson, N. J., Dichter, G. S., & Bodfish, J. W. (2012). Affective Responses by Adults with Autism Are Reduced to Social Images but Elevated to Images Related to Circumscribed Interests. *PLoS ONE*, *7*, e42457. https://doi.org/10.1371/journal.pone.0042457

Sasson, N. J., Turner‐Brown, L. M., Holtzclaw, T. N., Lam, K. S., & Bodfish, J. W. (2008). Children with autism demonstrate circumscribed attention during passive viewing of complex social and nonsocial picture arrays. *Autism Research*, *1*, 31-42.

Saygin, A. P., Wilson, S. M., Hagler, D. J., Bates, E., & Sereno, M. I. (2004). Point-light biological motion perception activates human premotor cortex. *Journal of Neuroscience*, *24*, 6181-6188.

[10.1523/JNEUROSCI.0504-04.2004](https://doi.org/10.1523%2FJNEUROSCI.0504-04.2004)

Schneider, K. N., Sciarillo, X. A., Nudelman, J. L., Cheer, J. F., & Roesch, M. R. (2020). Anterior cingulate cortex signals attention in a social paradigm that manipulates reward and shock. *Current Biology*, *30*, 3724-3735.

Scott-Van Zeeland, A. A., Dapretto, M., Ghahremani, D. G., Poldrack, R. A., & Bookheimer, S. Y. (2010). Reward Processing in Autism. *Autism Research*, *3*, 53–67. https://doi.org/Doi 10.1002/Aur.122

Sims, T. B., Neufeld, J., Johnstone, T., & Chakrabarti, B. (2014). Autistic traits modulate frontostriatal connectivity during processing of rewarding faces. *Social Cognitive and Affective Neuroscience*, *9*, 2010–2016. <https://doi.org/10.1093/scan/nsu010>

Slavich, G. M., Way, B. M., Eisenberger, N. I., & Taylor, S. E. (2010). Neural sensitivity to social rejection is associated with inflammatory responses to social stress. *Proceedings of the National Academy of Sciences*, 107, 14817–14822.<https://doi.org/10.1073/pnas.1009164107>

Spreckelmeyer, K. N., Krach, S., Kohls, G., Rademacher, L., Irmak, A., Konrad, K., Kircher, T., & Gründer, G. (2009). Anticipation of monetary and social reward differently activates mesolimbic brain structures in men and women. *Social Cognitive and Affective Neuroscience*, *4*, 158–165. https://doi.org/10.1093/scan/nsn051

Steiger, J. H. (1980). Tests for comparing elements of a correlation matrix. *Psychological Bulletin*, *87*, 245–251. <https://doi.org/10.1037/0033-2909.87.2.245>

Stijovic, A., Siegel, M., Kocan, A. U., Bojkovska, I., Korb, S., & Silani, G. (2024). Defining social reward: A systematic review of human and animal studies. *Psychological Bulletin*. <https://psycnet.apa.org/doi/10.1037/bul0000455>

Tennie, C., Frith, U., & Frith, C. D. (2010). Reputation management in the age of the world-wide web. Trends in Cognitive Sciences, 14, 482–488.<https://doi.org/10.1016/j.tics.2010.07.003>

Vouloumanos, A., Hauser, M. D., Werker, J. F., & Martin, A. (2010). The Tuning of Human Neonates’ Preference for Speech. *Child Development*, *81*, 517–527. https://doi.org/10.1111/j.1467-8624.2009.01412.x

Wake, S. J., & Izuma, K. (2017). A common neural code for social and monetary rewards in the human striatum. *Social Cognitive and Affective Neuroscience*, *12*, 1558–1564. https://doi.org/10.1093/scan/nsx092

Walther, D., & Koch, C. (2006). Modeling attention to salient proto-objects. *Neural Networks*, *19*, 1395–1407. <https://doi.org/10.1016/j.neunet.2006.10.001>

Werner, N. S., Kerschreiter, R., Kindermann, N. K., & Duschek, S. (2013). Interoceptive Awareness as a Moderator of Affective Responses to Social Exclusion. *Journal of Psychophysiology*, 27, 39–50.<https://doi.org/10.1027/0269-8803/a000086>

Wheelwright, S., Baron-Cohen, S., Goldenfeld, N., Delaney, J., Fine, D., Smith, R., Weil, L., & Wakabayashi, A. (2006). Predicting Autism Spectrum Quotient (AQ) from the Systemizing Quotient-Revised (SQ-R) and Empathy Quotient (EQ). *Brain Research*, *1079*, 47–56. https://doi.org/10.1016/j.brainres.2006.01.012

Wing, L. (1981). Asperger’s syndrome: A clinical account. *Psychological Medicine*, *11*, 115–129. <https://doi.org/10.1017/S0033291700053332>

Yoon, L., Kim, K., Jung, D., & Kim, H. (2021). Roles of the MPFC and insula in impression management under social observation. Social Cognitive and Affective Neuroscience, 16, 474–483.<https://doi.org/10.1093/scan/nsab008>

Zhang, D., Shen, J., Bi, R., Zhang, Y., Zhou, F., Feng, C., & Gu, R. (2022). Differentiating the abnormalities of social and monetary reward processing associated with depressive symptoms. *Psychological medicine*, *52*, 2080-2094.

Zink, C. F., Tong, Y., Chen, Q., Bassett, D. S., Stein, J. L., & Meyer-Lindenberg, A. (2008). Know Your Place: Neural Processing of Social Hierarchy in Humans. *Neuron*, *58*, 273–283. https://doi.org/10.1016/j.neuron.2008.01.025

# 
