# Supplementary material for: Autistic traits modulate neural and behavioral responses to social vs nonsocial rewards
Source: Personal Neurosci. 2025 Sep 4;8:e4. doi: 10.1017/pen.2025.10003 (PMC12450549; doi:10.1017/pen.2025.10003)
Supplement: Haffey et al. supplementary material 2 — Haffey et al. supplementary material [file S2513988625100035sup002.docx]

# Supplementary materials

### S1. Non-parametric correlations

To address concerns about outliers being unduly influential, non-parametric Spearman’s rank correlations were conducted on AQ with *sociality bias* (*r_s_*(35) = -.34, *p* = .019), AQ with LFG to ACC connectivity (*r_s_*(35) = -.32, *p* = .027), AQ with LAI to mOFC (r*_s_*(35) = -.3, p = .037), LFG to ACC with *sociality bias* (*r_s_*(35) = .44, *p* = .003) and LAI to mOFC with *sociality bias* (*r_s_*(35) = .47, p = .002).

## S2. Holm-Sidak corrected correlations between sociality bias and connectivity between seed and target regions.

| Seed | Target | Sociality (r) | Sociality (p) | outliers | Holm–Šidák threshold |
| --- | --- | --- | --- | --- | --- |
| LAmy | LVS | −0.08 | 0.67600 | 3 | 0.02532 |
| LAmy | RVS | −0.04 | 0.59227 | 3 | 0.01695 |
| LAmy | ACC | 0.08 | 0.32997 | 4 | 0.00851 |
| LAmy | mOFC | 0.46 | 0.00301 | 3 | 0.00233 |
| RAmy | LVS | −0.16 | 0.82061 | 1 | 0.05000 |
| RAmy | RVS | 0.16 | 0.17225 | 2 | 0.00465 |
| RAmy | ACC | 0.15 | 0.20392 | 3 | 0.00639 |
| RAmy | mOFC | 0.46 | 0.00308 | 3 | 0.00244 |
| LFG | LVS | 0.25 | 0.06888 | 1 | 0.00366 |
| LFG | RVS | 0.07 | 0.35069 | 3 | 0.01021 |
| **LFG** | **ACC** | **0.64** | **0.00004** | 5 | **0.00213** |
| LFG | mOFC | 0.06 | 0.37237 | 3 | 0.01274 |
| RFG | LVS | 0.16 | 0.18315 | 2 | 0.00512 |
| RFG | RVS | 0.23 | 0.09540 | 3 | 0.00394 |
| RFG | ACC | 0.41 | 0.00798 | 3 | 0.00270 |
| RFG | mOFC | 0.15 | 0.20232 | 3 | 0.00568 |
| LAI | LVS | 0.42 | 0.00628 | 2 | 0.00256 |
| LAI | RVS | 0.34 | 0.02208 | 2 | 0.00301 |
| LAI | ACC | 0.18 | 0.15092 | 2 | 0.00427 |
| **LAI** | **mOFC** | **0.52** | **0.00051** | 1 | **0.00223** |
| RTPJ | LVS | 0.30 | 0.03892 | 2 | 0.00341 |
| RTPJ | RVS | 0.35 | 0.02207 | 3 | 0.00285 |
| RTPJ | ACC | 0.08 | 0.31465 | 1 | 0.00730 |
| RTPJ | mOFC | 0.33 | 0.03092 | 4 | 0.00320 |

Results in bold are significant after correcting for multiple testing.
